# The role of *fruitless* in specifying courtship behaviors across divergent *Drosophila* species

**DOI:** 10.1126/sciadv.adk1273

**Published:** 2024-03-13

**Authors:** Christa A. Baker, Xiao-Juan Guan, Minseung Choi, Mala Murthy

**Affiliations:** Princeton Neuroscience Institute, Princeton University, Princeton, NJ, USA.

## Abstract

Sex-specific behaviors are critical for reproduction and species survival. The sex-specifically spliced transcription factor *fruitless* (*fru*) helps establish male courtship behaviors in invertebrates. Forcing male-specific *fru* (*fruM*) splicing in *Drosophila melanogaster* females produces male-typical behaviors while disrupting female-specific behaviors. However, whether *fru*’s joint role in specifying male and inhibiting female behaviors is conserved across species is unknown. We used CRISPR-Cas9 to force FruM expression in female *Drosophila virilis*, a species in which males and females produce sex-specific songs*.* In contrast to *D. melanogaster*, in which one *fruM* allele is sufficient to generate male behaviors in females, two alleles are needed in *D. virilis* females. *D. virilis* females expressing FruM maintain the ability to sing female-typical song as well as lay eggs, whereas *D. melanogaster* FruM females cannot lay eggs. These results reveal potential differences in *fru* function between divergent species and underscore the importance of studying diverse behaviors and species for understanding the genetic basis of sex differences.

## Teaser

Some functions of the highly conserved *fruitless* gene have diverged between a duetting and non-duetting *Drosophila* species.

## INTRODUCTION

Sex-specific behaviors, such as reproduction, aggression, and parental care, are essential for species survival. Many of these innate behaviors are under genetic control in both vertebrates and invertebrates (*1*). In many insects, sex-specific splicing of two transcription factors called *fruitless* (*fru*) and *doublesex* are responsible for establishing sexually differentiated neural circuitry and somatic tissue (*2*–*10*). In *Drosophila melanogaster*, in which *fru* function was first described (*11*), splicing in the male pattern results in a functional protein called FruM in a subset (~2000, or ~2%) of male neurons, whereas splicing in the female pattern results in transcripts that are degraded, leading to no functional protein (*3*, *12*, *13*). Sex-specific *fru* splicing has since been found across many but not all insect species (*14*–*22*). Whether the role of *fru* in specifying sex-specific behaviors differs across species remains an open question.

**Fig. 1.**
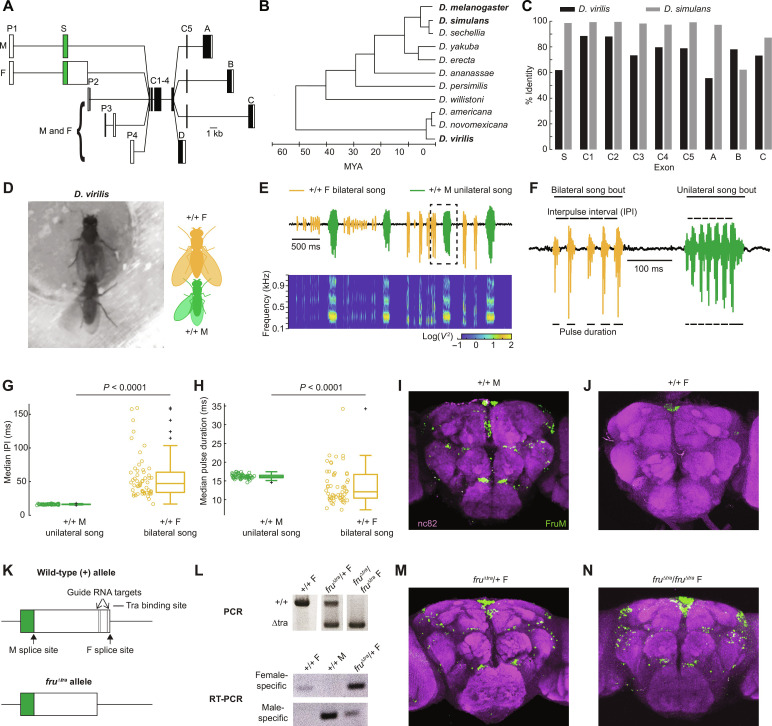
CRISPR-Cas9 editing of the *fruitless* (*fru*) gene results in expression of male-specific FruM in *D. virilis* female brains. **(A**) Transcripts resulting from alternative splicing of the *fru* gene. Filled and open boxes indicate coding and non-coding regions, respectively. P1 to P4 indicate promoters, S indicates the sex-specifically spliced exon, C1 to C5 indicate exons common to most *fru* transcripts, and A to D indicate alternative 3′ exons. Adapted from (*3*). (**B**) *Drosophila* phylogeny. Adapted from (*39*). (**C**) Comparison of the *fru* exon (coding regions only) nucleotide sequences. Percent identity is reported relative to *D. melanogaster*. (**D**) Video still (left) and schematic (right) of *D. virilis* courtship duets. Wild-type (+/+) *D. virilis* males and females sing using unilateral and bilateral wing vibration, respectively. (**E**) Microphone recording (top) and spectrogram (bottom) of an example duet. Songs were automatically segmented using a convolutional neural network (fig. S3; see Materials and Methods). (**F**) Close-up of the microphone recording outlined in (E). (**G** to **H**) IPI (G) and pulse durations (H) of unilateral and bilateral song. Each dot represents the median from one fly. Statistical tests were Wilcoxon rank sum tests with Bonferroni correction (*n* = 55 flies in both groups). (**I** and **J**) Antibody staining for FruM (green) and bruchpilot (nc82; magenta) in *D. virilis* +/+ male (I) and +/+ female (J) brains. The ocelli are FruM-immunopositive. (**K**) Schematic of the S-exon (top) and the result of CRISPR-Cas9–mediated removal of the transformer (Tra) binding sites (*fru*^Δ*tra*^) (bottom). (**L**) Top: Polymerase chain reaction (PCR) genotyping of *fru^Δ*tra*^* mutants using primers flanking the CRISPR guide RNA (gRNA) targets. Heterozygotes have both a +/+ and a shorter mutant allele (middle), whereas homozygous mutants have only the mutant allele (right). Bottom: Reverse transcription PCR (RT-PCR) using *D. virilis* female- and male-specific primers confirm that the brains of *fru^Δ*tra*^/+* females contain male *fru* transcripts. (**M** and **N**) FruM antibody staining in *D. virilis fru^Δ*tra*^/+* (M) and *fru^Δ*tra*^/fru^Δ*tra*^* (N) female brains. F, female; M, male.

Knocking out FruM expression in male *D. melanogaster* eliminates their ability to engage in courtship behaviors directed toward a female (*3*, *23*), and this function appears to be conserved in insects (*17*, *24*–*27*) [but see (*28*)]. Subsets of FruM-expressing neurons play distinct roles in producing male courtship behaviors in *D. melanogaster* (*5*, *29*–*33*), and at least some of these neurons are conserved across *Drosophila* species despite divergence in courtship behaviors (*23*, *34*, *35*). A breakthrough in our understanding of *fru* function came from forcing male-specific *fru* splicing in female *D. melanogaster* (*3*, *6*). This experiment was critical because *fruM* transcripts are also alternatively spliced at the 3′ end (Fig. 1A), giving rise to three isoforms differentially expressed across neurons: FruM-A, FruM-B, and FruM-C (*3*, *33*, *36*, *37*). Females with male-specific splicing of *fruM* not only made FruM protein but also expressed the correct isoform in each cell type. Unexpectedly, these females engaged in male courtship behaviors, with disruptions in their ability to produce female-specific behaviors; hence, *fru* was considered a “switch gene” for specifying sexually dimorphic behaviors (*3*). Here, we use a similar strategy (via CRISPR-Cas9 gene editing) to force FruM expression in females of another *Drosophila* species and investigate whether this also results in male-typical courtship behaviors while impeding female-typical behaviors. In other words, does FruM expression in females result in different outcomes depending on the species context?

We selected *Drosophila virilis*, which has male-specific FruM expression (*22*, *38*) but which diverged from *D. melanogaster* almost 60 million years ago (Fig. 1B) (*39*) and shows only ~50 to 90% sequence identity (Fig. 1C). Importantly for our experiments, *D. virilis* produces markedly divergent courtship behaviors compared to *D. melanogaster.* Courtship in *D. melanogaster* consists of the male pursuing the female while tapping, licking, and singing to her with unilateral wing extensions (*40*). Females, in turn, arbitrate mating decisions by slowing down and allowing copulation when receptive and then laying eggs. In the vast majority of drosophilid species with courtship songs, it is the male who sings to the female (*41*). Females have been reported to sing back to males in just a few species, all within the *D. virilis* group (*42*). *D. virilis* males use unilateral wing vibration, while females use bilateral wing vibration to produce sex-specific pulse songs during acoustic duets (Fig. 1, D and E) (*43*). Males can also sing a female-like bilateral song on the infrequent occasions when they are courted by another male (*43*), demonstrating that song choice is context-dependent in males. In contrast, females do not naturally produce the male-typical unilateral song. Therefore, unilateral song is male-specific, whereas bilateral song appears to be sexually monomorphic in *D. virilis*.

Here, we analyze potential evolutionary divergence and conservation of the role of *fru* between *D. virilis* and *D. melanogaster*. Although FruM expression in *D. virilis* females results in male courtship behaviors, including unilateral song production, these effects require two alleles of *fruM* in *D. virilis* but just one allele in *D. melanogaster* (*3*). FruM expression in *D. virilis* females alters the amount and sound features of bilateral song produced while duetting with a male; such a function was not possible to query in *D. melanogaster* because those females do not sing. Similar to *D. melanogaster*, FruM expression reduced receptivity in *D. virilis* females, but, in contrast to *D. melanogaster*, FruM expression did not eliminate egg laying in *D. virilis* females. Whereas pairings between wild-type males and FruM females were dominated by courtship in *D. melanogaster*, *D. virilis* FruM females alternated between duetting and male-directed aggression. These results highlight the value of comparing diverse behaviors and species in evaluating the role of important “switch genes” involved in behavioral specification.

## RESULTS

In *D. virilis* duets, two key features distinguish male-typical unilateral from female-typical bilateral song: the time between successive pulses, called interpulse intervals (IPIs), and the duration of each pulse (Fig. 1F). These features are stereotyped within and across males but are more variable in females (Fig. 1, G and H). In addition to unilateral song, *D. virilis* males can sing the female-typical bilateral song if they are courted by another male (fig. S1, A to C) (*43*), although male-directed courtship occurs less frequently than female-directed (fig. S1D). Male and female bilateral song is similar (fig. S1, E to G), suggesting that *D. virilis* may have two song circuits: one sexually monomorphic circuit for bilateral song and one dimorphic circuit for unilateral song. The role of *fru* in establishing either of these song circuits is unknown.

### Transformer binding site removal results in FruM expression in *D. virilis* female brains

Similar to *D. melanogaster*, FruM expression in *D. virilis* is male-specific (Fig. 1, I and J) (*22*, *38*). To understand the role FruM plays in specifying *D. virilis* courtship behaviors, we forced *fruM* splicing in female brains by removing the Transformer (Tra) binding sites from the sex-specifically spliced S exon (Fig. 1K) using CRISPR-Cas9, which in *D. melanogaster* results in splicing at the male site (*3*). This mutation is equivalent to the *fru*^Δ*tra*^ mutation in *D. melanogaster* (*3*). Polymerase chain reaction (PCR) (Fig. 1L, top) and sequencing confirmed that our mutagenesis resulted in removal of a portion of the S-exon containing the Tra binding sites. Reverse transcription PCR (RT-PCR) against female- and male-specific *fru* transcripts confirmed that *fru*^Δ*tra*^/+ female brains contained both versions (Fig. 1L, bottom), demonstrating that Tra binding site removal was sufficient to produce male-specific *fru* transcripts. We then validated these results with antibody staining for FruM (see Materials and Methods) and found that females carrying the *fru*^Δ*tra*^ mutation had FruM expression in subsets of neurons (Fig. 1, M and N) that overall was consistent with the +/+ male expression pattern (Fig. 1I). In particular, we were able to identify all eight FruM^+^ neuron clusters described in the anterior central brain of *D. melanogaster* (fig. S2A) and at least four of the eight posterior clusters (fig. S2B). However, the relatively low resolution (20×) of these images prevented us from comparing the numbers of FruM^+^ cells across genotypes. Therefore, while the *D. virilis fru*^Δ*tra*^ alleles result in FruM expression in female brains, we are not able to assess potential differences in the level of FruM expression and/or number of FruM^+^ neurons.

### One copy of *fru^Δ*tra*^* is insufficient for male courtship behaviors in *D. virilis* females

**Fig. 2.**
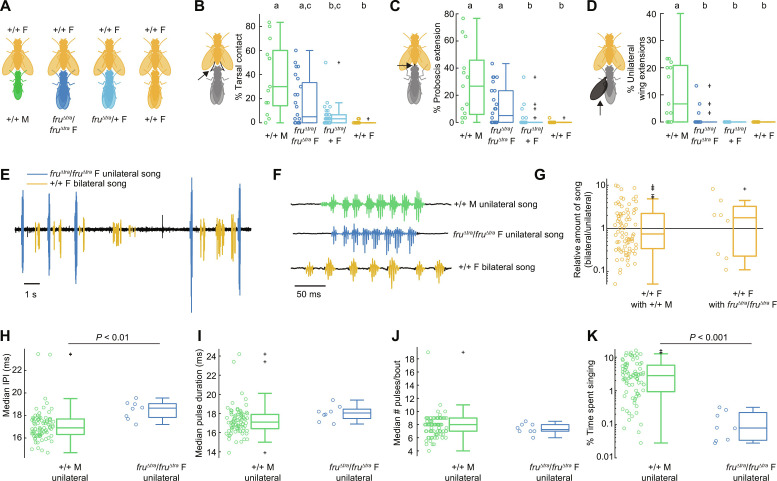
*D. virilis* *fru*^Δ*tra*^/*fru*^Δ*tra*^ females are capable of producing male-typical courtship behaviors directed toward +/+ females. (**A**) To test whether FruM specifies male courtship behaviors in *D. virilis*, we paired single *fru*^Δ*tra*^/*fru*^Δ*tra*^ and *fru*^Δ*tra*^/*+* females with a +/+ female. +/+ males and +/+ females (siblings to the *fru*^Δ*tra*^ females) each paired with a +/+ female served as controls. (**B** to **D**) % of manually scored bins containing instances where the experimental fly contacted the +/+ female with their front tarsi [(B), arrow in pictogram], extended their proboscis [(C), arrow], and extended wings unilaterally [(D), arrow]. Each dot represents the value for a single fly. Statistical significance was determined by Kruskal-Wallis tests followed by pairwise Wilcoxon rank sum tests with Bonferroni correction (*n* = 14, 24, 39, and 14 flies). (**E**) Audio recording (15 s) of a duet between a *fru*^Δ*tra*^/*fru*^Δ*tra*^ female and a +/+ female. (**F**) Close-up of the waveforms of +/+ male unilateral, *fru*^Δ*tra*^/*fru*^Δ*tra*^ female unilateral, and +/+ female bilateral songs. (**G**) Amount of bilateral song produced by the +/+ female normalized by the amount of unilateral song produced by the +/+ male (left) and *fru*^Δ*tra*^/ *fru*^Δ*tra*^ female (right). Each dot represents one pair of flies. (**H** to **K**) Median IPI (H), median pulse duration (I), median number of pulses per bout (J), and percent time each fly spent singing unilateral song (K). Statistical tests were Wilcoxon rank sum tests with Bonferroni correction [*n* = 83 and 8 flies in (G) to (K)].

The effects of FruM expression in *D. melanogaster* females requires only one copy of the *fru*Δ*tra* allele (*3*), a pattern known as dominance. This suggests that the amount of FruM transcription factor resulting from one *fru*^Δ*tra*^ allele is sufficient to produce masculinized neural circuitry in females. To determine whether *fruM* is also dominant in *D. virilis*, we paired a single *fru*Δ*tra*/+ female with a wild-type female (Fig. 2A) and quantified courtship behaviors. *D. virilis* courtship consists of the male orienting to the female, rubbing the underside of her abdomen with his front tarsi, licking her genitalia, and singing with unilateral wing extensions (*43*). We found that *D. virilis fru*^Δ*tra*^/+ females exhibited very little male-specific courtship behavior (light blue in Fig. 2, B to D), and no unilateral song in pairings with wild-type females (Fig. 2D). These results differ from those in *D. melanogaster* females, in which one allele of *fru*^Δ*tra*^ leads to male courtship behaviors (*3*).

In contrast to *D. melanogaster* (*3*), *D. virilis fru*^Δ*tra*^/+ females produced offspring after copulating with males; we found that *fru*Δ*tra*/ *fru*Δ*tra* females courted wild-type females (movie S1), with no significant differences in the amount of tarsal contact or proboscis extension compared to wild-type males (Fig. 2, B and C). We also observed unilateral wing extensions in a few *fru*^Δ*tra*^/*fru*Δ*tra* females (Fig. 2D), suggesting that *fru*Δ*tra*/*fru*Δ*tra* females may sing unilateral song when paired with a female.

To quantify song production, we built a new *D. virilis* song segmenter consisting of two convolutional neural networks (see Materials and Methods): one trained to distinguish among the four signal classes (unilateral song, bilateral song, overlap, and no song) (fig. S3A) and a second trained to distinguish between two signal classes (bilateral song and no song) (fig. S3B). Combining the output of the two networks (fig. S3C) resulted in high precision and sensitivity for detecting both unilateral and bilateral song, with equally good performance across genotypes (fig. S3, D to F). The performance of this segmenter is superior to that previously developed for *D. virilis* duets (*43*).

We found unilateral song in 8 of the 24 (33%) pairings between *fru*^Δ*tra*^/*fru*Δ*tra* *D. virilis* females and wild-type females. This song alternated with bilateral song from the wild-type female (Fig. 2E) and occurred in stereotyped bouts that looked similar to wild-type male unilateral song (Fig. 2F). We visually confirmed that the unilateral song occurred when the *fru*^Δ*tra*^/*fru*Δ*tra* female was performing unilateral wing extensions and that the bilateral song was produced solely by the wild-type female (movie S1). Wild-type females sang just as much bilateral song with *fru*Δ*tra*/*fru*Δ*tra* females as with wild-type males (Fig. 2G). *fru*Δ*tra*/*fru*Δ*tra* unilateral song had short IPIs in line with those of wild-type males, although the IPI was modestly but significantly increased by 1 to 2 ms (Fig. 2H). There was no difference in pulse duration (Fig. 2I) or the number of pulses per bout (Fig. 2J) between the unilateral song from *fru*^Δ*tra*^/*fru*Δ*tra* females and wild-type males. However, we found that *fru*Δ*tra*/*fru*Δ*tra* females produced almost an order of magnitude less unilateral song than wild-type males (Fig. 2K). Together, these results reveal that, while FruM arising from the *fru*Δ*tra* allele specifies male-typical unilateral song in *D. virilis*, it is not sufficient (even with two alleles) to produce male-typical levels of courtship drive. Because FruM is a transcription factor involved in the development of sexually dimorphic neural circuitry, this finding suggests that, although *fru*Δ*tra*/*+* and *fru*Δ*tra*/*fru*Δ*tra* *D. virilis* females developed some FruM^+^ neurons (Fig. 1, M and N), there may be important differences in FruM expression levels and/or FruM^+^ neuron number, morphology, function, or connectivity patterns dependent on allele number.

We next returned to *D. melanogaster* to reinvestigate *fruM* allele number and song production. We conducted single-pair courtship experiments using two *D. melanogaster* *fru*Δ*tra* genotypes (fig. S4A): *fru*Δ*tra*/+ to match the genotype of the *D. virilis* *fru*Δ*tra*/+ females, and *fru*Δ*tra*/*fru^4-40^* (where *fru^4-40^* is a fruM-null mutation) (*12*) to compare with previous experiments (*3*, *44*). Both *fru*Δ*tra*/+ (movie S2) and *fru*Δ*tra*/*fru^4-40^ D. melanogaster* females courted wild-type females. Whereas both *fru*^Δ*tra*^/+ and *fru*^Δ*tra*^/*fru^4-40^* females engaged in tapping and unilateral wing extensions directed toward a wild-type female (fig. S4, B to D), the amount of these behaviors were significantly less than those produced by wild-type males (fig. S4, C and D). Consistent with prior work (*44*), we found that, while *fru*^Δ*tra*^ females extended their wings, this did not produce bonafide song (fig. S4, E and F).

Together, our results reveal two key species differences in the role of FruM in specifying male courtship behaviors: (i) One copy of a *fru*^Δ*tra*^ allele can produce male courtship in *D. melanogaster* females but not in *D. virilis* females; (ii) two alleles of *fru*Δ*tra* produce male-typical unilateral song in female *D. virilis*, whereas the effects of two alleles of *fruM* cannot be tested in female *D. melanogaster* as one allele renders females infertile (*3*). We are not able to rule out whether these species differences could be due to differences in FruM expression levels resulting from *D. virilis* versus *D. melanogaster* *fru*Δ*tra* alleles.

### Removing a *fruM* allele in *D. virilis* males has no effect on courtship behaviors

The requirement of two *fruM* alleles to produce unilateral song in *D. virilis* females raised the question of whether a similar pattern occurs in males. Wild-type males make (via splicing) two functional copies of FruM RNA in each cell, one from each allele. What happens to male behavior if we remove one of these copies? We generated males lacking one copy of *fruM* by removing the S-exon (fig. S5A) via CRISPR-Cas9 and confirming the removal with PCR (fig. S5, B and C) and sequencing. We refer to these males as −/+. We then paired −/+ males with wild-type females (fig. S5D) and found that −/+ males robustly courted females. There was a modest reduction in tarsal contact by −/+ males compared to wild-type males (fig. S5E) but no differences in the amount of proboscis extension (fig. S5F) or unilateral wing extension (fig. S5G). Wild-type females duetted with −/+ males, and the waveforms of −/+ male unilateral song appeared similar to wild-type male unilateral song (fig. S5, H to J). We found no differences in the quantitative parameters of −/+ unilateral song or in the amount or timing of unilateral song (fig. S5, K to O). −/+ males were also as likely to copulate as wild-type males (fig. S5P). Together, these results demonstrate that two copies of *fruM* are needed for the production of male courtship behaviors, including unilateral song, only in the female context in *D. virilis*.

### FruM disrupts female receptivity but not egg laying in *D. virilis*

**Fig. 3.**
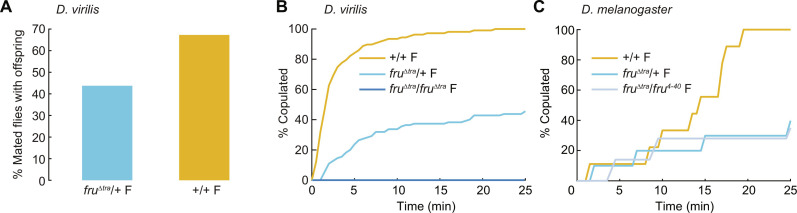
Effects of FruM expression on female reproductive behaviors of *D. virilis* and *D. melanogaster*. (**A**) Percentage of matings with +/+ males that resulted in larvae in *D. virilis*. *n* = 48 and 107 flies. (**B**) Cumulative percentage of copulated *D. virilis* females (paired with +/+ conspecific males) over the 25-min observation period. Curves are normalized to +/+ females. *n* = 118, 121, and 24 flies. (**C**) Same as (B) for *D. melanogaster*. *n* = 34, 38, and 54 flies.

FruM expression in female *D. melanogaster* not only results in male-typical courtship behaviors but also interferes with female-typical courtship behaviors, such as egg laying and receptivity (*3*). In contrast to *D. melanogaster*, *D. virilis* *fru*^Δ*tra*^*/+* females maintained their ability to lay eggs, as almost 50% of *fru*^Δ*tra*^*/+* females that copulated produced offspring (Fig. 3A). In our single-pair courtship assays, the copulation rate of *D. virilis* *fru*^Δ*tra*^*/+* females was about 40% that of wild-type females (Fig. 3B). In a notable similarity between species, the copulation rate of *D. melanogaster* *fru*^Δ*tra*^ females was also 35 to 40% that of wild-type females (Fig. 3C) within our 25-min observation period. These results point to divergent effects of FruM expression on egg laying but conserved effects on female receptivity between the two species.

Adding a second allele of *fru*^Δ*tra*^ to *D. virilis* females completely eliminated copulation (Fig. 3B) within our 25-min assays. This was not due to reduced attractiveness to males, as males directed equal amounts of courtship behaviors toward females of all genotypes (fig. S6, A to C). Therefore, FruM effects on female receptivity in *D. virilis* depend on the number of *fruM* alleles.

### FruM alters female-typical bilateral song features and amount

**Fig. 4.**
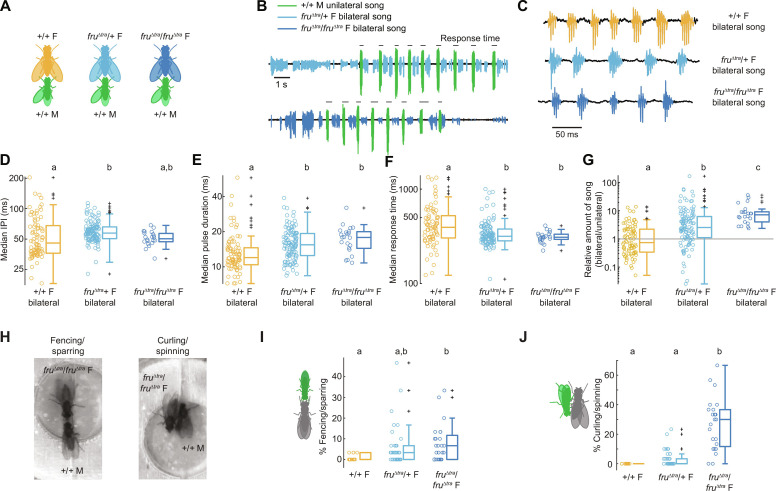
FruM expression in *D. virilis* females has effects on bilateral song and aggression. (**A**) To determine whether FruM expression affected bilateral song production, we paired single *fru*^Δ*tra*^/+ and *fru*^Δ*tra*^/*fru*^Δ*tra*^ *D. virilis* females with a +/+ male. Single +/+ females (siblings of the *fru*^Δ*tra*^ females) paired with a +/+ male served as controls. (**B**) Microphone recordings (15 s) of duets between *fru*^Δ*tra*^ females singing bilateral song and +/+ males singing unilateral song. In these pairings, unilateral song was produced solely by the +/+ male and bilateral song solely by the female. (**C**) Close-up of bilateral song waveforms. (**D** to **G**) Bilateral song median IPI (D), pulse duration (E), response time (delay between onset of unilateral bout and center of first following bilateral pulse) (F), and amount relative to unilateral song (G). Each dot represents one fly. *n* = 83, 114, and 22 flies. Statistical tests were Kruskal-Wallis tests followed by pairwise Wilcoxon rank sum tests with Bonferroni correction. (**H**) Video stills of fencing/sparring (left) and curling/spinning (right). (**I** to **J**) Percentage of bins with fencing/sparring (I) and curling/spinning (J). Each dot represents one fly. *n* = 13, 38, and 24 flies.

The effects of FruM expression on female singing behavior has not previously been tested in any species. Because *D. virilis* bilateral song is sexually monomorphic (fig. S1) (*43*), we expected FruM expression in females to have no effect on bilateral song production. In pairings of *fru*^Δ*tra*^/+ and *fru*^Δ*tra*^/*fru*^Δ*tra*^ *D. virilis* females with wild-type males (Fig. 4A), *fru*^Δ*tra*^ females readily duetted with wild-type males (Fig. 4B and movie S3), with the female singing solely bilateral song and the male singing solely unilateral song. The waveform of *fru*Δ*tra* bilateral song looked similar to wild-type female bilateral song (Fig. 4C). However, one allele of *fru*^Δ*tra*^ resulted in increased IPIs by about 10 ms relative to wild-type females (Fig. 4D). FruM expression also led to longer pulses (Fig. 4E) and shorter response times (Fig. 4F), regardless of the number of *fru*^Δ*tra*^ alleles. Unexpectedly, FruM expression markedly increased the amount of bilateral song, with *fru*^Δ*tra*^/*fru*^Δ*tra*^ females singing almost an order of magnitude more bilateral song than wild-type females when paired with a wild-type male (fig. S6D). Because bilateral song is produced during a partnered duet, we wondered whether this increase in *fru*^Δ*tra*^ bilateral song simply reflected increased levels of courtship. However, wild-type males sang less unilateral song with a *fru*^Δ*tra*^/*fru*^Δ*tra*^ female (fig. S6E) and directed equal amounts of other courtship behaviors toward *fru*^Δ*tra*^ and wild-type females (fig. S6, A to C). Accounting for differing amounts of unilateral song from the male revealed a significant increase in the amount of bilateral song produced by *fru*^Δ*tra*^ females (Fig. 4G). Increased bilateral song in *fru*^Δ*tra*^ females is consistent with the increased bilateral song produced by males relative to wild-type females when courted by another male (fig. S1H), suggesting that an up-regulation of bilateral song may be a consequence of FruM-induced masculinization. Together, our results show that, although bilateral song is sexually monomorphic, at least some of the underlying neural circuitry may be regulated by *fru*.

### Homozygous *fru*^Δ*tra*^ *D. virilis* females display male-directed aggression

We observed two types of male-directed aggressive behaviors from *D. virilis fru*^Δ*tra*^ females (Fig. 4H). Male-directed aggression was not reported in *fru*^Δ*tra*^ *D. melanogaster* females (*3*, *6*), because these pairings are dominated by courtship from the male (*45*). In one behavior, the *fru*^Δ*tra*^ *D. virilis* female and male face each other and extend their front tarsi toward one another (movie S4), similar to previously described “low-posture fencing” (*46*) or “sparring” (*47*). In the second behavior, the *fru*^Δ*tra*^ female curls her abdomen toward the male’s head, similar to previously described curling (*47*, *48*). While the female is curling, the male still tries to tap and lick her abdomen, which results in the two flies spinning together in a circle (movie S5). These aggressive behaviors were interspersed with duetting, with duetting often immediately preceding and/or following an aggressive bout. The amount of fencing/sparring and curling/spinning behaviors was dependent on the number of *fru*^Δ*tra*^ alleles in females (Fig. 4, I and J).

*fru*^Δ*tra*^/*fru*^Δ*tra*^ *D. virilis* females were the most likely to produce aggression (Fig. 4, I and J) and were also least likely to copulate (Fig. 3B). However, we found no difference in the amount of aggressive behaviors in copulated versus non-copulated *fru*^Δ*tra*^/+ females (fig. S6, F and G). These behaviors do not seem to be typical reactions to courtship of an unreceptive female, because non-copulating wild-type females did not engage in these behaviors (Fig. 4, I and J). We also did not observe aggressive behaviors in pairings between two wild-type males (dark green in fig. S5, Q to R), and only rarely between −/+ males and wild-type males (light green in fig. S5, Q to R), suggesting that these behaviors are dependent on the number of *fruM* alleles in *D. virilis* females but not males.

Although we and others did not observe aggression from *D. melanogaster* FruM females paired with wild-type males, likely because these pairings are dominated by female-directed courtship, *fru* does play a role in specifying sex-specific aggression in *D. melanogaster* males and females (*45*, *49*–*51*). Together, these results illustrate an additional species difference: In *D. melanogaster*, pairings between wild-type males and *fru*^Δ*tra*^ females result in primarily female-directed courtship, whereas similar pairings in *D. virilis* result in alternations between female-directed courtship and male-directed aggression.

### *fru*^Δ*tra*^ effects on courtship and aggression depend on allele number in female, but not male, *D. virilis*

**Fig. 5.**
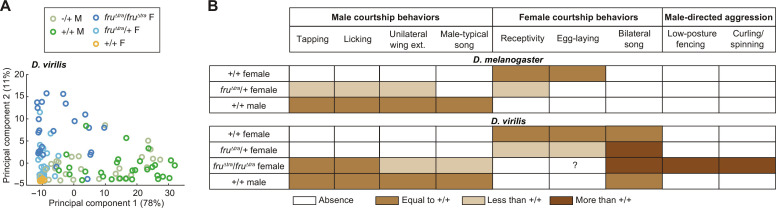
FruM effects on *D. virilis* courtship and aggressive behaviors depend on allele number in females. (**A**) Principal components analysis (PCA) on all scored courtship and aggressive behaviors exhibited by a single fly when paired with a +/+ female and (separately) a +/+ male. Principal component 1 (PC1) positively correlated with tarsal contact and proboscis extension directed toward a +/+ female, and PC2 positively correlated with curling/spinning and tarsal contact, both directed toward a +/+ male. Each dot represents one fly. *n* = 26 −/+ M, 29 +/+ M, 24 *fru*^Δ*tra*^/*fru*^Δ*tra*^ F, 39 *fru*^Δ*tra*^*/+* F, and 14 +/+ F flies. (**B**) Table summarizing the effects of FruM expression in *D. melanogaster* (top) and *D. virilis* (bottom). Colors indicate the amount of each behavior.

So far, our results suggest that, in female *D. virilis*, FruM effects on both unilateral and bilateral song production as well as aggression depend on the number of *fru*^Δ*tra*^ alleles. To clarify the effects of FruM in both sexes, we performed principal components analysis (PCA) on courtship and aggressive behaviors exhibited by the same fly paired with a wild-type female versus a wild-type male (Fig. 5A). These results show that, compared to wild-type females, which do not produce any aggressive or male-typical courtship behaviors, adding one copy of *fru*^Δ*tra*^ to females caused movement primarily along principal component 2 (PC2; Fig. 5A), which correlated positively with both aggression (curling/spinning) and courtship (tarsal contact) directed toward a wild-type male. A second *fru*^Δ*tra*^ allele caused females to also move along PC1 (Fig. 5A), which correlated positively with courtship (tarsal contact and proboscis extension) directed toward a wild-type female. Only a few of the *fru*^Δ*tra*^/*fru*^Δ*tra*^ females overlapped in PCA space with wild-type males, suggesting that FruM, while sufficient to produce male courtship behaviors including unilateral song in some *D. virilis* females, is not sufficient to fully masculinize females. In contrast to the effects of *fruM* allele number in females, removing one *fruM* allele in males had no effect on courtship or aggression (Fig. 5A). These results lend further support to the conclusion that *fruM* has allele number–dependent effects in *D. virilis* females but not males*.*

## DISCUSSION

Here, we provide evidence for both similarities and differences in the function of *fru* between two *Drosophila* species*.* We were able to make these comparisons by using CRISPR-Cas9 to force native male-specific *fruM* splicing in female *D. virilis*, which had previously been accomplished only in *D. melanogaster*. One of the notable species differences that we found is that *D. virilis*, but not *D. melanogaster*, females expressing FruM maintain their fecundity (Fig. 5B), enabling us to test the effects of one versus two alleles of *fru*^Δ*tra*^ on behaviors in *D. virilis*, but not in *D. melanogaster*. This revealed that *fruM* allele number in females (but not in males) affected the phenotypes observed. In contrast to *D. melanogaster*, in which one copy of *fru*^Δ*tra*^ is sufficient to produce some male courtship behaviors, two copies were needed in *D. virilis* (Fig. 5B). These male courtship behaviors were accompanied by male-typical song production in female *D. virilis* but not *D. melanogaster* (Fig. 5B) (*3*, *44*). Therefore, while FruM plays a role in male-typical unilateral wing vibrations in both species, the extent to which the resulting song is fully masculinized is different across species. FruM expression in female *D. virilis* also had opposing effects on receptivity and bilateral (female-typical) song amount (Fig. 5B); effects on female song production could not be assayed in *D. melanogaster* because those females do not sing. That FruM expression does not inhibit egg laying or bilateral song production in *D. virilis* is different from the role of FruM in suppressing female behaviors in *D. melanogaster* and *Aedes aegypti* (*3*, *24*). However, the disruption of female receptivity in FruM *D. virilis* females is in line with similar results in *D. melanogaster*. Last, *D. virilis* females expressing FruM exhibited male-directed aggression, whereas *D. melanogaster* females did not (Fig. 5B).

### A role for FruM in both unilateral and bilateral song production in *D. virilis*

We found that FruM expression conferred some *D. virilis* females with the ability to produce male-specific (female-directed) unilateral song while up-regulating the amount of (male-directed) bilateral song. This suggests that the circuitry underlying each song type is susceptible to FruM expression and also raises the possibility that overlapping (instead of distinct) neural populations may contribute to unilateral and bilateral song in *D. virilis* males. Although *D. melanogaster* females do not naturally produce song, mutations of genes outside the sex determination cascade have caused females to produce unilateral wing extensions (*52*, *53*). Artificial activation of *fru*^+^ ventral nerve cord circuitry in female *D. melanogaster* produced unilateral wing extensions with aberrations in pulse and sine song that were ameliorated by FruM expression (*54*). Activation of the *dsx*^+^ pC1 brain neuron cluster also produced male song in females (*55*). Together, these findings suggest that female *D. melanogaster* has latent circuitry capable of song production and that FruM unlocks the ability of this circuitry to produce male courtship behaviors, including wing extensions. It is tempting to speculate that the processes involved in producing male-specific behaviors in FruM female *D. melanogaster* might be similar to the processes involved in the up-regulation of bilateral song in FruM female *D. virilis.*

### Differences between behaviors of *fru*^Δ*tra*^/*+* and *fru*^Δ*tra*^/*fru*^Δ*tra*^ *D. virilis* females

In *D. virilis*, we found differences in the amount of male courtship behaviors, bilateral song, aggression, and receptivity between females that were heterozygous versus homozygous for *fru*^Δ*tra*^. This pattern is suggestive of haploinsufficiency, in which one copy of a gene is insufficient for a particular phenotype. Haploinsufficiency is not uncommon for transcription factors. For instance, *fruM* is haploinsufficient for pheromone responses in Or47b olfactory receptor neurons in male *D. melanogaster* (*56*). In mice, haploinsufficiency of the transcription factor *Six3* disrupts male reproduction by impeding development of the main olfactory epithelium (*57*). In human sex determination, multiple transcription factors display haploinsufficiency leading to sex reversals (*58*). Causes of haploinsufficiency are hypothesized to include insufficient amounts of protein product arising from a single gene copy, stoichiometric disruptions of protein complexes, and, more recently, a narrow range of tolerable protein expression levels (*58*, *59*). However, homozygous *fru*^Δ*tra*^ *D. virilis* females were not fully masculinized, as only a few of these flies produced male-like levels of courtship behaviors. This result is likely due to the interplay between *fru* and other sex-determination genes, such as *doublesex* (see the next section)*.*

Our finding that two copies of *fru*^Δ*tra*^ are needed for male courtship behaviors in female *D. virilis* is different from the results of similar experiments in *D. melanogaster*, in which one copy of *fru*^Δ*tra*^ produces male-specific behaviors (*3*). One cause of this apparent species difference could be that our *D. virilis* *fru*^Δ*tra*^ allele is hypomorphic for FruM, such that *fru*^Δ*tra*^/+ *D. virilis* females express less FruM protein relative to wild-type males than *fru*^∆*tra*^/+ *D. melanogaster* females. This would mean that *fru*^Δ*tra*^/+ *D. virilis* female brains express enough FruM to reduce female receptivity but not enough to disrupt egg laying or to display male courtship behaviors, suggesting potential dose-dependent effects of FruM in *D. virilis* females. Quantification of FruM protein levels in *fru*^Δ*tra*^ females of both species would be required to determine how FruM expression levels may relate to behavioral phenotypes.

### Differences between behaviors of *D. virilis* males and *fru*^Δ*tra*^ females

We found that *fruM* allele–dependent effects were specific to the female context in *D. virilis*, because normal male courtship behavior was observed with just one allele of *fruM* in males (fig. S5). Another transcription factor called *doublesex* (*dsx*) is also sex-specifically spliced in *D. melanogaster* and, in contrast to *fru*, produces functional protein in males (DsxM) and females (DsxF) (*2*). Dsx is expressed in subsets of neurons that play key roles in both male- and female-specific behaviors (*5*, *60*–*63*) and is co-expressed with FruM in some cell types in male brains (*5*, *36*, *44*, *64*). Females generally have fewer numbers of *dsx*^+^ and *fru*^+^ neurons than males due to DsxF-dependent cell death (*65*) and FruM-dependent inhibition of cell death (*66*). Therefore, while the presence of FruM in female *Drosophila* brains sufficiently masculinizes some cell types (*67*), DsxF may act to limit the extent of this masculinization. For instance, although some *fru*^Δ*tra*^ females of both species produced male courtship behaviors, including singing, the overall levels of these behaviors were lower than those of wild-type males. Because of DsxF-mediated cell death (*5*), *D. melanogaster* FruM females lack the male-specific and DsxM^+^/FruM^+^ P1 neurons, which play a critical role in male courtship initiation and persistence (*33*, *68*, *69*). Therefore, the lower levels of male courtship produced by FruM females relative to males is in line with the absence of P1 neurons.

### Role of FruM in *D. virilis* aggression

We found that, in pairings with wild-type males, *fru*^Δ*tra*^ *D. virilis* females alternate between playing the female-typical role in acoustic duets and directing aggression toward the male. We only saw these behaviors in *fru*^Δ*tra*^ females, raising the concern that these are aberrant behaviors caused by partial masculinization in a female background. Multiple lines of evidence argue against this possibility. First, curling and fencing/sparring have previously been reported in wild-type *D. virilis* (*47*, *48*), suggesting that these are species-typical behaviors. It is possible that we did not provide sufficient conditions, such as a food patch or competing male, to provoke these behaviors in wild-type males. Second, although we did not observe male-directed aggression from *D. melanogaster fru*^Δ*tra*^ females, these females do show aggression in other contexts (*45*), and subsets of FruM^+^ neurons play a role in generating aggression in *D. melanogaster* (*49*–*51*). Therefore, a role for FruM in aggression in *D. virilis* is consistent with a similar role in *D. melanogaster*, although the species-specific types of aggressive behaviors may be different. The aggression of *fru*^Δ*tra*^ *D. virilis* females is unlikely to be a rejection of male courtship, as non-copulating females did not produce more of these behaviors than copulating females. Males also were not deterred by these behaviors and instead would often resume courtship immediately following an aggressive bout. In almost all instances, the *fru*^Δ*tra*^ female was the initiator of the aggression, and our interpretation of the spinning that accompanied the curling behavior is that the male was trying to reach the female’s abdomen and genitalia with his foretarsi and proboscis, respectively. As the female curled and spun around, the male followed. Together, these results suggest that FruM plays a role in producing species-typical aggression and that FruM expression in female *D. virilis* brains causes the female to be aggressive in situations where she otherwise would not be.

### Importance of analyzing the role of switch genes across species

Findings in other insect species of sex-specific *fru* splicing and/or disruption of male courtship behaviors after *fruM* knockdown led to the hypothesis that *fru*’s role as a sex-determination switch gene was highly conserved (*14*, *15*, *17*, *18*, *23*, *24*, *26*, *27*, *70*). This hypothesis was also supported by findings that inserting *fru* genes from drosophilid species with divergent courtship behaviors into *D. melanogaster* males recapitulated *D. melanogaster* behaviors, instead of phenocopying each species’ own behaviors (*71*), which suggested that divergence in FruM downstream targets likely contributes to specifying species-specific behaviors. However, in most species previously studied, males and females engage in markedly different behaviors during courtship. By choosing a species in which the sexes produce a similar behavior, i.e., singing, we uncovered potential differences in some aspects of *fru* function, despite conservation of sex-specific FruM expression (Fig. 1, I and J) (*22*, *38*). Additional sex-determination switch genes in *D. melanogaster* include *dsx* and three genes upstream of *fru* and *dsx*: *sex-lethal* (*sxl*), *transformer* (*tra*), and *transformer-2* (*tra2*). Whereas *dsx*, *sxl*, and *tra2* appear to be conserved at the sequence level in *D. virilis* (*2*, *72*, *73*), *tra* sequence comparisons suggest functional divergence in *D. virilis* and other *Drosophila* species (*74*, *75*). The extent to which sequence divergence in sex determination genes contributes to species-specific behaviors remains to be determined. Quantifying how these genes are expressed in individual cell types will be critical to evaluating potential divergence versus conservation of gene function at the circuit level. Our findings here of divergent effects of FruM expression on sex-specific behaviors in *D. virilis* highlight the importance of going beyond sequence comparisons in carefully selected species for evaluating conservation versus divergence of switch gene function.

In summary, through gene editing and careful behavior quantification, we found evidence for both differences and similarities in *fru* function in divergent *Drosophila* species. Future work should investigate *fru* circuitry underlying sex-specific behaviors across species to understand the neural basis of behavioral divergence.

## MATERIALS AND METHODS

### Fly strains

We used *D. virilis* 15010-1051.47 (*43*) and *D. melanogaster* NM91 as wild-type strains. *D. virilis* was kept on standard medium at 20°C on a 16-hour:8-hour light:dark cycle (*76*) and aged 10 to 20 days, as this is the time required to reach sexual maturity (*77*). *D. melanogaster* was kept on standard medium at 25°C on a 12-hour:12-hour light:dark cycle and aged 3 to 7 days. Flies expected to produce male courtship behaviors [i.e., males and *fru*^Δ*tra*^/*fru*^Δ*tra*^, *fru*^Δ*tra*^/*+*, and wild-type sibling (*D. virilis*) or control (*D. melanogaster*) females] were singly housed within 8 hours of eclosion, whereas courtship targets (wild-type females that were not siblings of *fru*^Δ*tra*^ females) were housed in groups of five to six flies.

### Comparison of *fruitless* nucleotide sequences

We downloaded the following data in April and May 2023: *D. melanogaster fru* (FBgn0004652) exon sequences from ensembl.org; the *D. virilis* scaffold (scaffold_12855) containing *fru* and the *Drosophila simulans* chromosome (ch3R) containing most *fru* exons from the UC Santa Cruz Genome Browser (https://genome.ucsc.edu/); and the sequences containing the *D. simulans* B (accession number GI: 111258132) and C (accession number: KF005597) exons from GenBank (*78*). We used Geneious Prime 2023.1.2 to align the nucleotide sequence of each *D. melanogaster* exon to the relevant *D. virilis* and *D. simulans* sequences and recorded the % identity.

### Generation of *D. virilis fruitless* mutants

#### *fru* ^Δ*tra*^

To examine the role of *fruitless* in *D. virilis* song production, we used CRISPR-Cas9 mutagenesis to remove the Tra binding sites from the S exon, similar to the *fru*^Δ*tra*^ mutation previously made in *D. melanogaster* (*3*). We identified the Tra binding sites in *D. virilis* based on sequence similarity with *D. melanogaster* (*79*). We then designed CRISPR guide RNAs (gRNAs) flanking the Tra binding sites using the CRISPR Optimal target finder (*80*). The gRNAs had a 20-nt target sequence and were flanked by a 3′ protospacer adjacent mofif (PAM) sequence (“NGG”) and a 5′ T7 RNA polymerase recognition sequence (“GG”). The target genomic region was sequenced using Sanger sequencing. gRNAs are listed below. The 5′ is the T7 promoter, bold indicates the gRNA target, and italics indicate the 3′ portion that overlaps with the reverse primer. The PAM is shown in parentheses.

L1: 5′ GAAATTAATACGACTCACTATA**GGTGTCTATGCCTAGGACTT**(AGG)*GTTTTAGAGCTAGAAATAGC 3*′

R1: 5′ GAAATTAATACGACTCACTATA**GGCTAGAGGCACGTGAGTAG**(TGG) *GTTTTAGAGCTAGAAATAGC 3*′

R2: 5′ GAAATTAATACGACTCACTATA**GGAACTGCATACCGTGCGGCA**(TGG) *GTTTTAGAGCTAGAAATAGC 3′*

The forward primer format and single guide RNA-reverse (sgRNA-R) primer sequences are based on (*81*). To generate the template for each sgRNA, we used the CRISPR forward and reverse 4 nmol Ultramer oligonucleotides (Integrated DNA Technologies). The full-length dsDNA template was amplified using Invitrogen Platinum PCR super mix high fidelity (catalog no.12532-016) and 0.5 μM forward and reverse primers. Reactions were carried on an Applied Biosystems 2720 Thermal Cycler, 95°C 2 m, 35 cycles of (95°C for 20 s, 60°C for 10 s, and 70°C for 10 s) and then purified with Ampure XP beads (A63880). In vitro transcription of 300 ng of sgRNA template DNA using T7 MEGAscript kit (Invitrogen, AM1333) was carried out at 37°C for 16 to 20 hours. Turbo deoxyribonuclease (Invitrogen, AM2239) was added for an additional 15 min at 37°C and then purified with Mega Clear Kit (Invitrogen, AM1908). The gRNA concentration and quality were checked with Agilent Bioanalyzer and frozen in small aliquots at −80°C for long-term storage. The CRISPR injection mixture contained recombinant Cas9 protein (300 ng/μl; PNA Bio CP01) and sgRNA (40 ng/ μl; per guide; we used one upstream L1 and two downstream R1 and R2 gRNA) and was injected into embryos of *D. virilis* wild-type strain 15010-1051.47. The Insect Transformation Facility at the University of Maryland performed all injections. We backcrossed the injected G0 flies to wild-type flies and selected lines in which germline mutagenesis was successful as determined by PCR genotyping (see below). PCR and sequencing confirmed that L1 and R2 successfully cut the DNA and removed 622 base pairs, including the Tra binding sites. We obtained 12 independent lines carrying the *fru*^Δ*tra*^ mutant allele and observed no differences in behaviors across lines.

### fruM-null

The method to remove the S-exon of the *fruitless* gene followed the same general procedure described for the *fru*^Δ*tra*^ design, with the following changes. We designed two CRISPR target sites (L-1 and L-3) upstream of the *D. virilis fru* S-exon start codon. The target sites downstream of the S-exon were as described earlier (R1 and R2).

L-1: GAAATTAATACGACTCACTATA**GGAAACCTTTAAACGGAGAAT**(TGG)*GTTTTAGAGCTAGAAATAGC*

L-3: GAAATTAATACGACTCACTATA**GGACCAACTAGTGCTAGAT**(CGG)*GTTTTAGAGCTAGAAATAGC*

The injection mix contained the L-1, L-3, R1, and R2 gRNA. We crossed injected flies to one another and used PCR genotyping of the offspring to identify lines with germline transmission. PCR and sequencing confirmed that the L-1 and R1 guides successfully removed the S-exon. We obtained seven independent lines in which the S-exon was removed.

### PCR genotyping

We used PCR to identify the genotype of experimental flies. We extracted DNA from the whole fly or from just the body (saving the heads for immunostaining from a subset of flies) using a Quick-gDNA miniprep kit (Zymo Research, R1050). We designed primers upstream and downstream of the Tra binding sites as follows:

CRISPRcut F3: TACGTACACGAATAGCCTCTTG

CRISPRcut R1: TGCCCGATTGAGCAAAATGC

We designed an additional primer upstream of the S-exon to identify flies in which the S-exon was successfully removed.

CRISPRcut F1: TGAGAGTTGTGTGATGGCTTG

### Reverse transcription polymerase chain reaction

We made total RNA from fly heads using a Quick RNA Microprep kit (Zymo Research, R1050). The reverse transcription reaction used the High-Capacity cDNA Reverse Transcription Kit (Applied Biosystems, 4368814) and RNase inhibitor (Promega, N2515). We designed male-specific and female-specific forward RT-PCR primers and a primer from a downstream *fru* common exon based on sequence similarity with those used previously in *D. melanogaster* (*8*).

Female-specific primer 10136 Fwd: GCAAAAGGAAGAGAGC-CTCA

Male-specific primer 8200 Fwd: GATGGCCACCTCACAAGATT

Common primer C4 Rev: GCAGTCCATATTTCGAGACGA

### Immunohistochemistry

We dissected brains in ice-cold phosphate-buffered saline (PBS) and then fixed in 4% paraformadehyde in PBSX1 (CellGro, 21-040) with 0.3% Triton X-100 (Sigma-Aldrich, X100) (abbreviated PBT3) for 45 min in the dark at room temperature. We blocked with 5% normal goat serum (Life Technologies, 16210-064) in PBT3 for two overnights at 4°C. We incubated brains with 1:5000 rabbit anti-FruM (*36*) (gift from S. Goodwin) and 1:20 mouse anti-nc82 (Developmental Studies Hybridoma Bank, Iowa City, Iowa) in blocking solution for three overnights at 4°C. We rinsed eight times for 20 min in PBT3 at room temperature before an overnight incubation in 1:500 goat anti-rabbit Alexa Fluor 633 (Invitrogen, A21070) and 1:500 goat anti-mouse Alexa Fluor 488 (Life Technologies, A11001) in blocking solution at 4°C. We rinsed four times for 20 min in PBT3 and then four times for 20 min in PBS at RT before leaving brains overnight in Vectashield (Vector Laboratories, H-1000-10) at 4°C. We mounted brains in Vectashield and imaged on a confocal microscope (Leica TCS SP8 X). Images were adjusted for brightness and contrast in ImageJ (National Institutes of Health).

### Behavioral assays

Virgin males and females were used for behavior experiments. Video was captured at 60 frames/s with a Point Grey Flea 3 CMOS camera (FL3-U3-13E4C-C). Audio was recorded at 10 kHz on a 32-channel apparatus (*43*, *82*). Each experimental fly was paired with a single homosexual +/+ partner on day 1 and with a single heterosexual +/+ partner on day 2. Each recording lasted 25 or 30 min. Experimental flies were singly housed to maintain their identity for PCR genotyping and/or checking for larvae. All assays occurred at ~22°C and between 0 and 4 hour of Zeitgeber time (ZT) (*D. virilis*) or 0 to 1.5 hours of ZT (*D. melanogaster*). Circular behavioral chambers were scaled for differences in fly body sizes between species: 11-mm diameter for *D. melanogaster* (*82*) and 20-mm diameter for *D. virilis* (*43*).

### Behavior quantification

We uniformly sampled each video and manually scored 10-s bins spaced 1 min apart for the presence or absence of the following behaviors: tarsal contact (front tarsi contact with any part of the other fly); proboscis extension; unilateral wing extension; bilateral wing extension; wing-flicking (unilateral wing extension to ~45° that is quickly retracted without producing sound); fencing/sparring (flies face one another and extend their front tarsi toward each other while in a normal standing posture) (*46*); curling (fly curls abdomen laterally with the tip of the abdomen pointed toward the other fly) (*47*); and spinning (flies face in opposite directions and jointly move in a circle). Curling and spinning co-occurred so frequently that we did not attempt to score them separately. We report the percentage of bins containing at least one instance of the respective behavior in Figs. 2 (B to D) and 4 (I and J) and figs. S4 (C and D), S5 (E to G and Q to R), and S5 (A to C and F to G). We performed PCA on the amount of these seven behaviors each fly produced when paired with a +/+ male and a +/+ female using MATLAB 2019b. Copulation was defined as the time when the male first mounted the female and remained in the copulation position for at least 1 min in *D. virilis* (copulation duration, ~2 to 5 min) or 5 min in *D. melanogaster* (copulation duration, ~10 to 20 min) (*83*).

### *D. virilis* song segmenter

#### Network structure

The *D. virilis* song segmenter consisted of two convolutional neural networks in Python 3.4 (fig. S2, A and B): one network for classifying each point in the microphone recording as unilateral song, bilateral song, overlap of the two song types, or no song; and a second network for classifying bilateral song versus no song. Using the four-class network alone led to many bilateral song false positives, as noises such as grooming, jumping, and rolling were often classified as bilateral song. Therefore, to improve the precision of bilateral song detection, we used a second, two-class network to classify bilateral versus no song. Both networks take the raw microphone recording as input. The four-class network used a window size of 4001 points (400.1 ms), batch size of 128, six epochs, 16 convolutional filters, 2 × 2 pooling, a convolutional kernel size of 9 convolutions and 4 padding, and trained on 10% of the data. The two-class network used a similar structure as the four-class network except it used a window size of 2001 points (200.1 ms) and three epochs during training.

#### Training data

A single observer used both audio and video to manually segment five 30-min recordings of +/+ males paired with +/+ females and one 30-min recording of a +/+ male paired with a *fru*^Δ*tra*^/+ female. The *fru*^Δ*tra*^/+ female recording was chosen because of the large amount of song from both flies. We then drew from these data to make training data for the two neural networks. Training data for the four-class network consisted of 31.6 total min (28 min from the *fru*^Δ*tra*^/+ and +/+ male pairing and the remaining from the +/+ male and female pairs), resulting in a total of 4.9% unilateral song (585 unilateral bouts), 25.4% bilateral song (10,358 *fru*^Δ*tra*^/+ bilateral pulses, 92 +/+ bilateral pulses), and 1.3% overlap between unilateral and bilateral. We found the best performance when data from different rounds of data collection were included in the training set. To make the training data for the two-class network, we removed unilateral and overlap portions from the training data for the four-class network and added 21.5 min of additional noise and quiet taken from recordings of +/+ female pairs.

#### Song segmentation

To combine the output of the two segmenters, we first applied a median filter (window size of 10 ms) to the output probabilities and then averaged the bilateral and no song probabilities from the four- and two-class networks. We ignored the output of the two-class network during portions identified as unilateral or no song by the four-class network (fig. S3C). The maximum probability at each timepoint was used to identify instances of song, with the following heuristics. To classify a point as bilateral song, we required the bilateral probability to be at least 1.25 times that of unilateral song, and, to classify a point as overlap, we required the overlap probability to be at least 0.85. We required bilateral song within 40 ms before or up to 10 ms after unilateral song to have a classification probability of 1; otherwise, it was assigned to unilateral song. We threw out segments predicted to be bilateral if they were shorter than 4 ms. We next defined unilateral bouts as contiguous unilateral predictions and bilateral pulses as contiguous bilateral predictions. To separate contiguous unilateral or bilateral song into pulses, we first calculated the difference of the upper and lower peak envelopes of the microphone signal with a width of 5 ms and then detected peaks in this signal. Minima on either side of these peaks were used to define where one pulse ended and the next started. We compared the segmenter output to ground truth data obtained by manually segmenting 22 recordings (fig. S3, D to F). Sensitivity, positive predictive value, and the harmonic mean (*F*) were calculated as previously described (*82*).

#### Song analysis

To calculate IPIs, we first calculated the difference of the upper and lower peak envelopes of the microphone signal with a width of 5 ms and then detected peaks in this signal. The difference in timing of these peaks was taken as the IPI. Remaining song analysis was largely based on a previous study of *D. virilis* duets (*43*). A unilateral bout was defined as a series of at least four unilateral pulses with IPIs of 60 ms or less. A bilateral bout was defined as any number of bilateral pulses with IPIs of 100 ms or less. Unilateral response times were defined as the delay between the offset of a bilateral pulse and the onset of the immediately following unilateral bout. Bilateral response times were defined as the time between the onset of a unilateral bout and the center of the following bilateral pulse. Only response times less than 1.5 s were included in our analysis.

For song feature measurements, we used only recordings with at least 10 unilateral and 10 bilateral pulses before copulation in copulating pairs or over the entire recording otherwise. We quantified the amount of unilateral and bilateral song by summing the total time of the recording classified as unilateral or bilateral and then dividing by the courtship time, defined as the time between the first and last pulse (of either song type) in the recording (for non-copulating pairs) or the last pulse of either song type before copulation (for copulating pairs).

#### Statistics

Because most measurements were not normally distributed according to Jarque-Bera tests, we used nonparametric tests throughout. All statistics were performed in MATLAB 2019b or 2022a.

## Supplementary Materials

### This PDF file includes:

Figs. S1 to S6 Legends for movies S1 to S5 References
