# Supplementary material for: The role of fruitless in specifying courtship behaviors across divergent Drosophila species
Source: Sci Adv. 2024 Mar 13;10(11):eadk1273. doi: 10.1126/sciadv.adk1273 (PMC10936877; doi:10.1126/sciadv.adk1273)
Supplement: Supplementary file 1 — Figs. S1 to S6 Legends for movies S1 to S5 References [file sciadv.adk1273_sm.pdf]

Science Advances
AAAS

Supplementary Materials for

The role of *fruitless* in specifying courtship behaviors across divergent *Drosophila* species

Christa A. Baker *et al.*

Corresponding author: Mala Murthy, mmurthy@princeton.edu; Christa A. Baker, cbaker5@ncsu.edu

Sci. Adv. **10**, eadk1273 (2024)
DOI: 10.1126/sciadv.adk1273

The PDF file includes:

Figs. S1 to S6
Legends for movies S1 to S5
References

Other Supplementary Material for this manuscript includes the following:

Movies S1 to S5

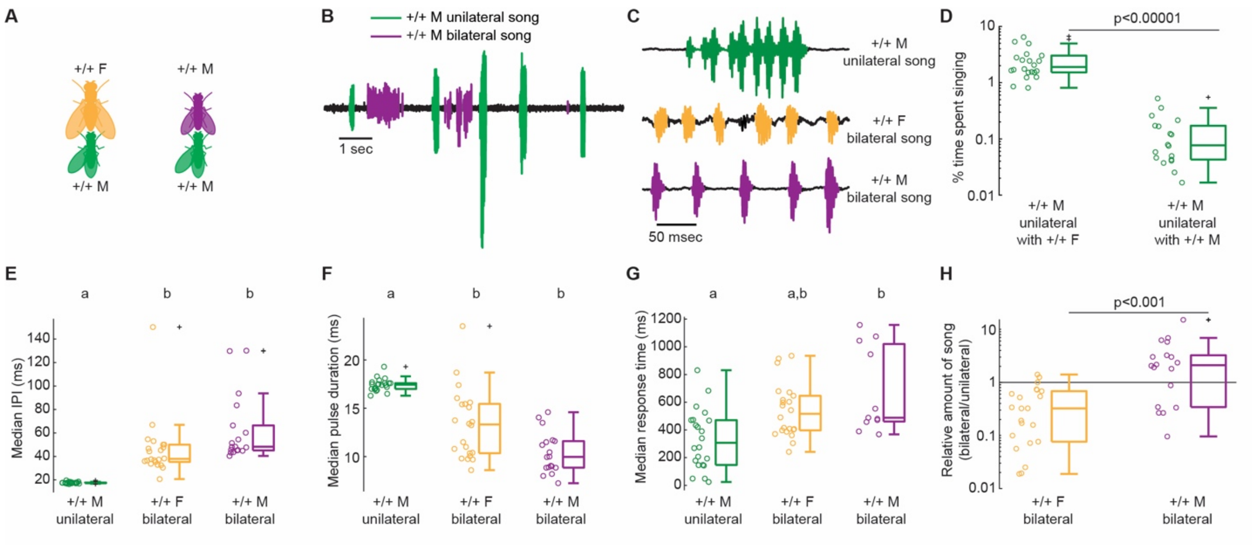

Fig. S1. *D. virilis* bilateral song is sexually monomorphic. (**A**) To compare bilateral song produced by +/+ males and females, we recorded song from heterosexual (left) and homosexual (right) pairs. (**B**) +/+ males are capable of singing bilateral song when courted by another male. (**C**) Bilateral song pulses from +/+ males are similar in appearance to +/+ female bilateral song and distinct from the stereotyped bouts of +/+ male unilateral song. (**D**) Total % of courtship time, defined as the time between the first and last pulse (from either sex) of each recording, that contains unilateral song. Although +/+ males take turns courting one another, the amount of male-directed courtship, as measured by the amount of unilateral song, is significantly lower than female-directed (Wilcoxon rank sum z=5.4, p<1e-7). (**E-G**) IPI (**E**), pulse duration (**F**), and response time (delay between onset of unilateral bout and center of first following bilateral pulse) (**G**) of +/+ male bilateral song compared to +/+ male unilateral and +/+ female bilateral songs. Each dot represents the within-fly median. Kruskal-Wallis and pairwise Wilcoxon rank sum tests with Bonferroni correction were used to detect significant differences between groups. (**H**) +/+ males sing more total bilateral song than +/+ females relative to the amount of unilateral song in a given recording (Wilcoxon rank sum z=-3.7, p<1e-3). n=11-22 flies/group in (**D-H**).

A

Anterior brain

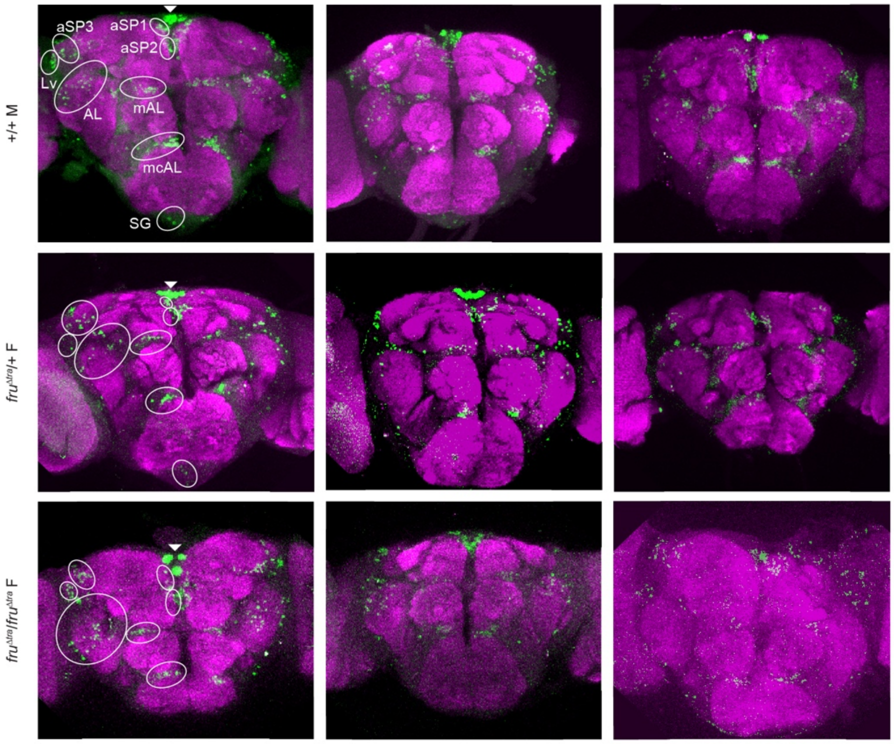

B

Posterior brain

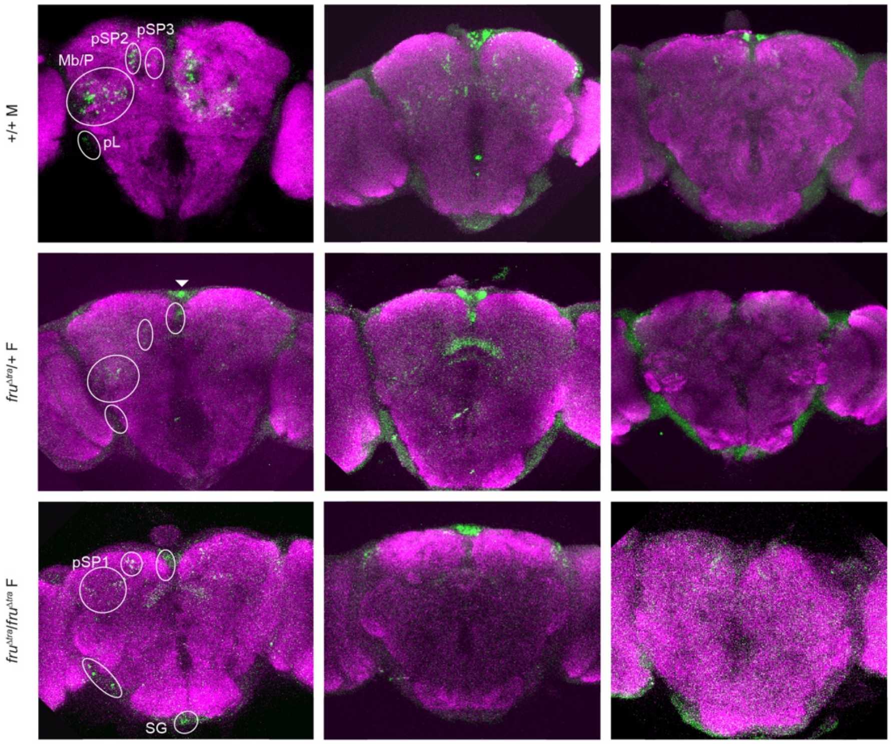

Fig. S2. FruM expression in *D. virilis* $fru^{\Delta\ tra}$/+ and $fru^{\Delta\ tra}/fru^{\Delta\ tra}$ female brains. (**A**) Antibody staining for FruM (green) and bruchpilot (nc82; magenta) in anterior *D. virilis* +/+ male (top), $fru^{\Delta\ tra}$/+ female (middle), and $fru^{\Delta\ tra}/fru^{\Delta\ tra}$ female brains. Three individuals from each genotype are shown. Clusters corresponding to FruM+ cell types in *D. melanogaster* (as defined in (*84*)) are indicated. We were able to identify all eight anterior FruM+ clusters in the central brain of *D. virilis*. Somas corresponding to the SG cluster in $fru^{\Delta\ tra}/fru^{\Delta tra}$ females appeared in more posterior sections than in $fru^{\Delta\ tra}$/+ females and +/+ males and are thus indicated in (**B**). (**B**) Same as (**A**) for the posterior portions of the same brains. In general, posterior staining was more faint than anterior, and thus even in +/+ *D. virilis* males, we were only able to identify 4-5 of the 8 FruM+ clusters described in posterior *D. melanogaster* brains (*84*). FruM+ cluster names follow those labeled in the top row unless otherwise indicated. Ocelli (arrowheads) are immunoreactive for FruM. The faintness of FruM staining in the posterior brain resulted in a low signal to noise ratio, such that optimizing visualization of FruM+ somas required contrast and brightness adjustments that make the protocerebral bridge appear in the green channel in a few brains.

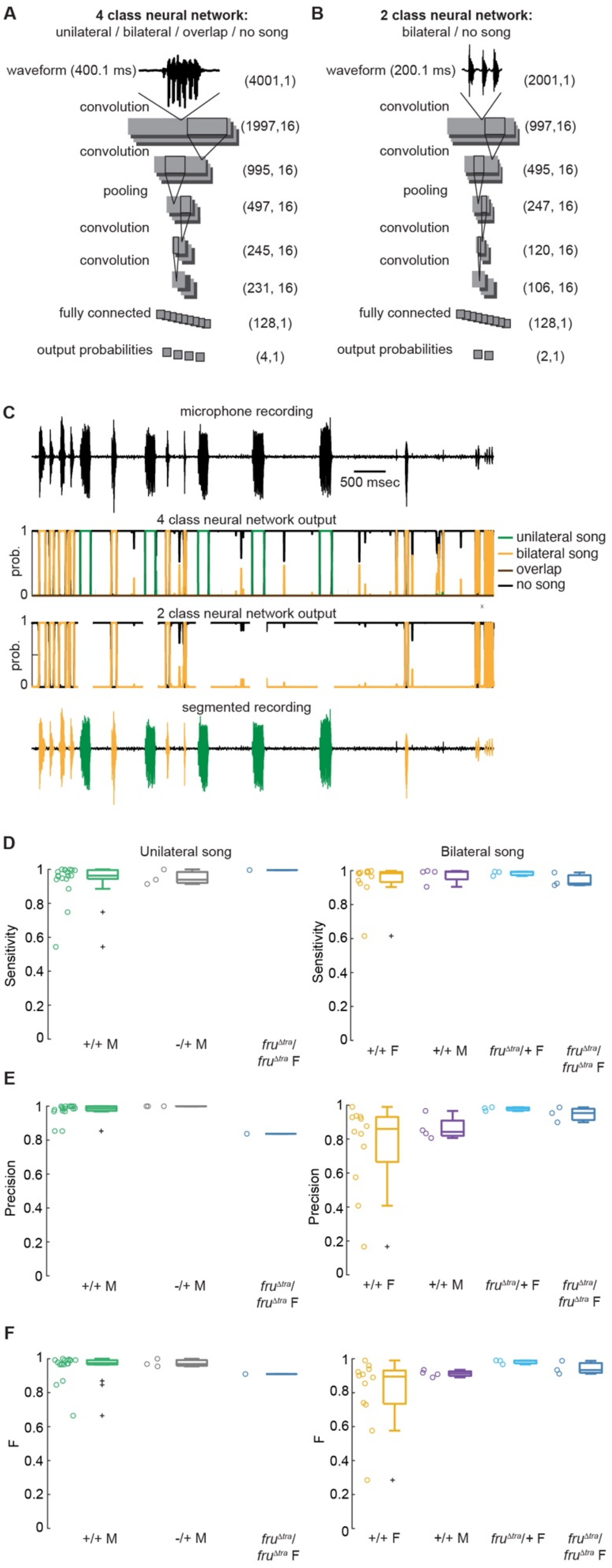

A
4 class neural network:
unilateral / bilateral / overlap / no song
waveform (400.1 ms)
(4001,1)
convolution
(1997,16)
convolution
(995, 16)
pooling
(497, 16)
convolution
(245, 16)
convolution
(231, 16)
fully connected
(128,1)
output probabilities
(4,1)
B
2 class neural network:
bilateral / no song
waveform (200.1 ms)
(2001,1)
convolution
(997,16)
convolution
(495, 16)
pooling
(247, 16)
convolution
(120, 16)
convolution
(106, 16)
fully connected
(128,1)
output probabilities
(2,1)
C
microphone recording
500 msec
4 class neural network output
prob.
unilateral song
bilateral song
overlap
no song
2 class neural network output
segmented recording
D
Unilateral song
Bilateral song
Sensitivity
+/+ M
-/+ M
fru^Δtra/ fru^Δtra F
+/+ F
fru^Δtra/+ F
E
Precision
F
F

Fig. S3. Convolutional neural network (CNN)-based song segmenter for *D. virilis* songs. (**A**) Architecture of the CNN trained to distinguish between unilateral song, bilateral song, overlap of the two song types, and no song. The input is a raw microphone recording with a window size of 400.1 ms, and the output is a series of classification probabilities for each time point in the recording. This network was good at identifying unilateral song and overlap portions, but often classified noises, such as jumping or grooming, as bilateral song. (**B**) For this reason we trained a second network to specifically distinguish between bilateral song and no song. This 2-class CNN was similar to the 4-class network (**A**) except it used a smaller window size of 200.1 ms. (**C**) Example of song segmenter pipeline. Determinations of unilateral song and overlap (which is rare) come from the 4-class network, and thus those portions of the recording are ignored in the output of the 2-class network. The classification probabilities for bilateral song and no song are averaged between the two networks. The segmenter assigns each point in the recording according to the maximum classification probability, with a few heuristics (see Materials & Methods). (**D-F**) Sensitivity (**D**), precision (**E**), and their harmonic mean F (**F**) of the segmenter performance on unilateral (left) and bilateral (right) song compared to manual segmentations. n=18, 3, 1 (left) and 12, 4, 3, 3 (right) flies.

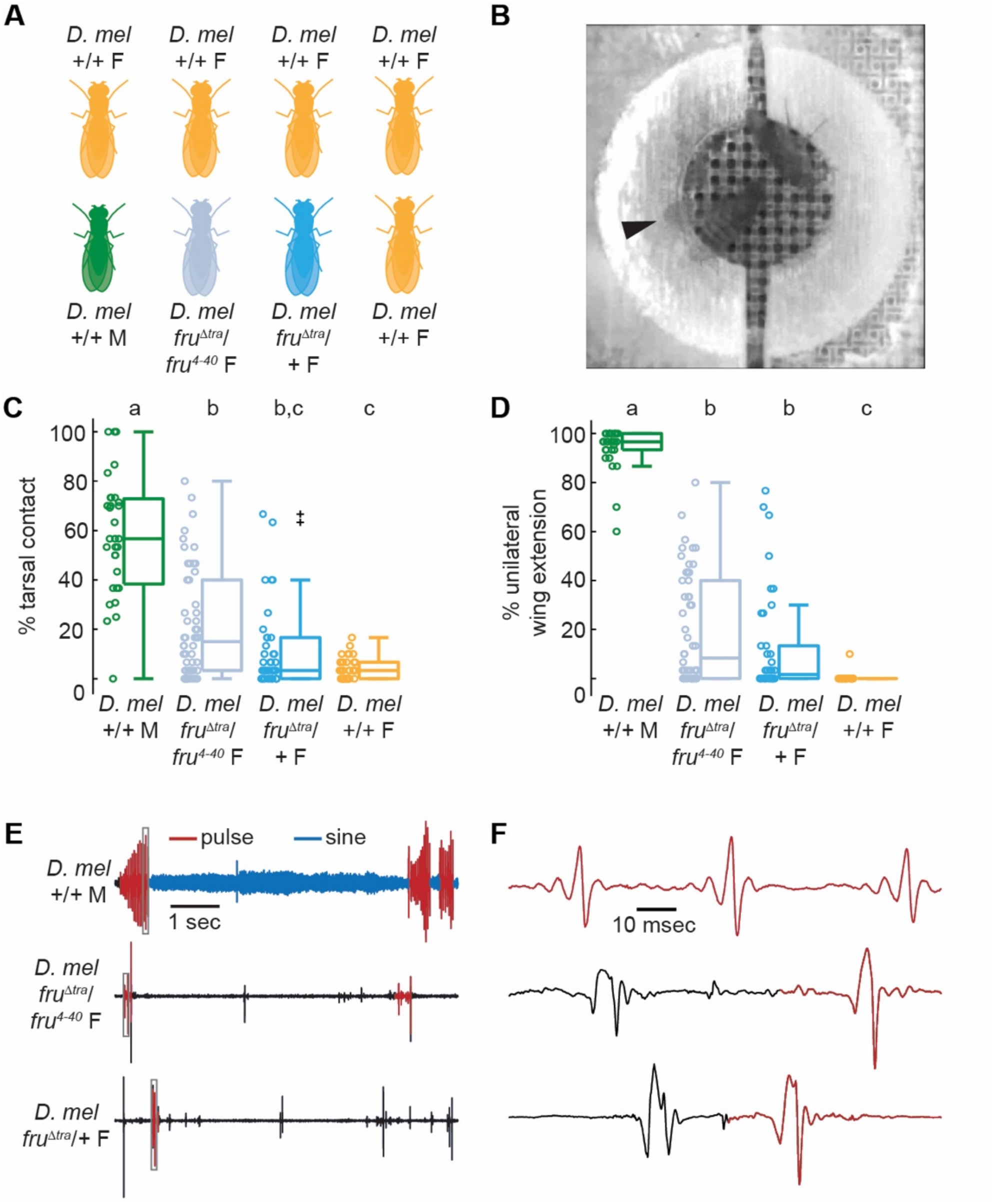

Fig. S4. ***D. melanogaster fru$^{\Delta tra}$* females produce male courtship behaviors but aberrant unilateral song.** (**A**) We paired single *fru$^{\Delta tra}$/fru$^{4\text{-}40}$* and *fru$^{\Delta tra}$*/+ *D. melanogaster* females with a +/+ (NM91) female. Single +/+ males and females each paired with a +/+ female served as controls. (**B**) A *fru$^{\Delta tra}$*/+ female performs unilateral wing extensions (arrowhead) directed toward a +/+ female. (**C-D**) Percentage of bins containing tarsal contact (**C**) or unilateral wing extensions (**D**) produced by each genotype when paired with a +/+ female. Each dot represents one fly. n=31, 54, 38, 34 flies. (**E**) Seven-second microphone recordings showing sounds concurrent with unilateral wing extensions directed toward a +/+ female. Unilateral wing extensions by males generate complex song bouts consisting of switches between pulse and sine song, whereas unilateral wing extensions by *fru$^{\Delta tra}$* females only infrequently generate pulses

(never sine). Some of these pulses are detected by the *D. melanogaster* song segmenter (red) (*82*). (**F**) Enlargements of the boxed regions in (**E**). *fru*$^{\Delta tra}$ female pulses detected by the *D. melanogaster* segmenter (red) lack the structure of +/+ male pulses (top).

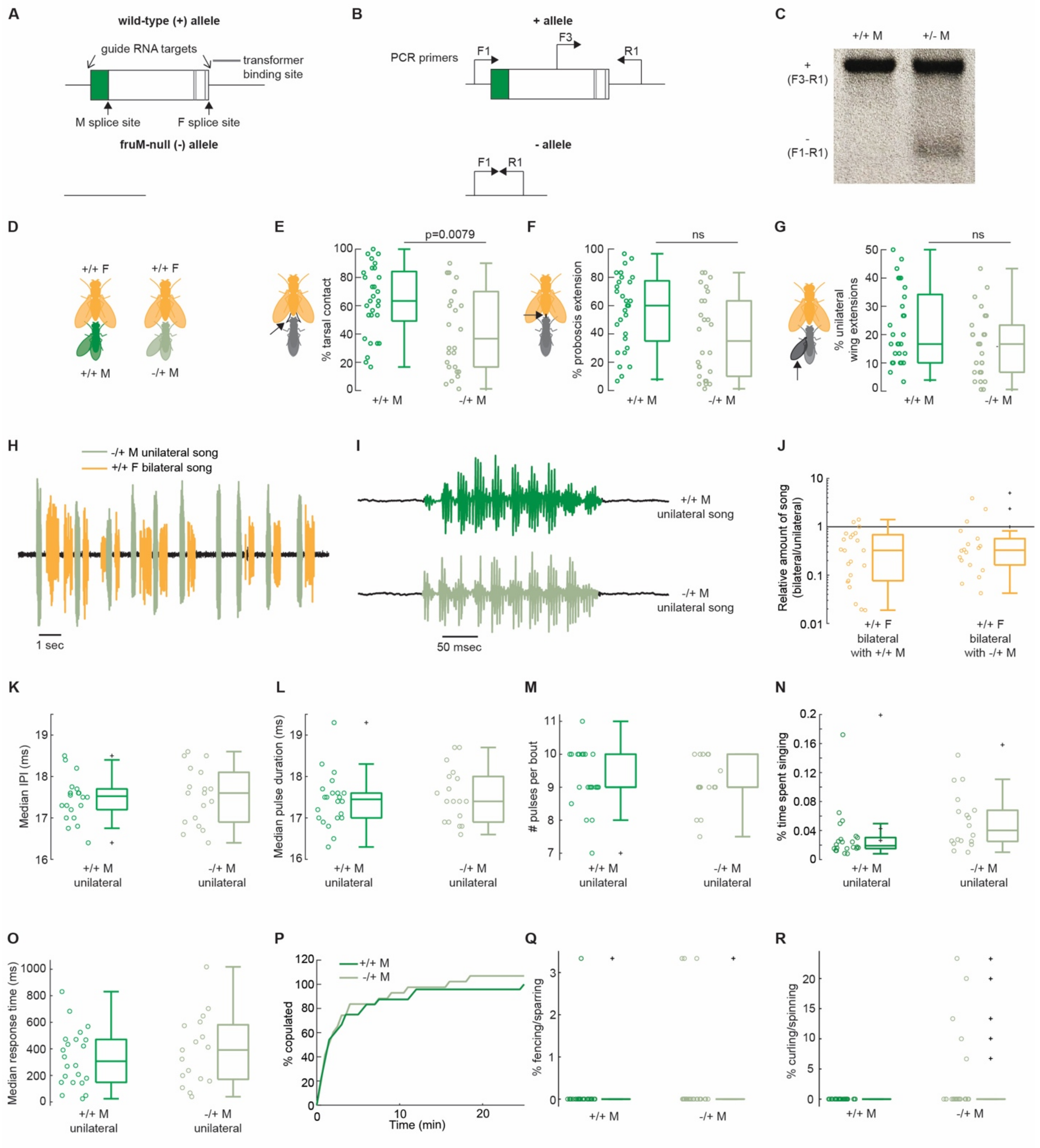

Fig. S5. Removing a copy of *fruM* from *D. virilis* males has no effect on courtship behaviors. (**A**) To generate a *fruM*-null allele, we designed CRISPR-Cas9 guide RNAs flanking the S-exon (top) to remove the S-exon (bottom). (**B**) We confirmed deletion of the S-exon with PCR using 1 reverse primer (R1) and 2 forward primers (F1, F3) due to the size of the S-exon. (**C**) PCR results using the primers shown in (**B**). In +/+ flies, the F1-R1 product is too large to amplify, so the product present is F3-R1 (left). The same product is present in -/+ flies due to the wild-type allele, in addition to the F1-R1 product, which is shorter due to the removal of the S-exon (right).

(**D**) To test whether removal of a *fruM* copy affected male courtship behaviors, we paired single -/+ males with a +/+ female. Single +/+ males (siblings to -/+ males) paired with a +/+ female served as controls. (**E-G**) Percent of bins containing tarsal contact (**E**), proboscis extension (**F**), and unilateral wing extension (**G**) directed toward a +/+ female. Each dot represents one fly. n=29 and 26 flies. (**H**) 14 sec microphone recording of a duet between a -/+ male and +/+ female. (**I**) A single unilateral song bout from a +/+ and a -/+ male. (**J-O**) Amount of bilateral song (from +/+ female) relative to unilateral song (from male) (**J**), median IPI (**K**), median pulse duration (**L**), median number of pulses per bout (**M**), percent time spent singing (**N**), and median response time (delay between offset of bilateral pulse and onset of following unilateral bout) (**O**) of unilateral song from each genotype when paired with a +/+ female. Each dot represents one fly. n=22 and 18 flies in (**J-O**). (**P**) Cumulative percent copulation over the 25 min observation period. Values are normalized relative to +/+ males. (**Q-R**) Percent bins with fencing/sparring (**Q**) and curling/spinning (**R**) behaviors. n=29 and 26 flies in (**P-R**).

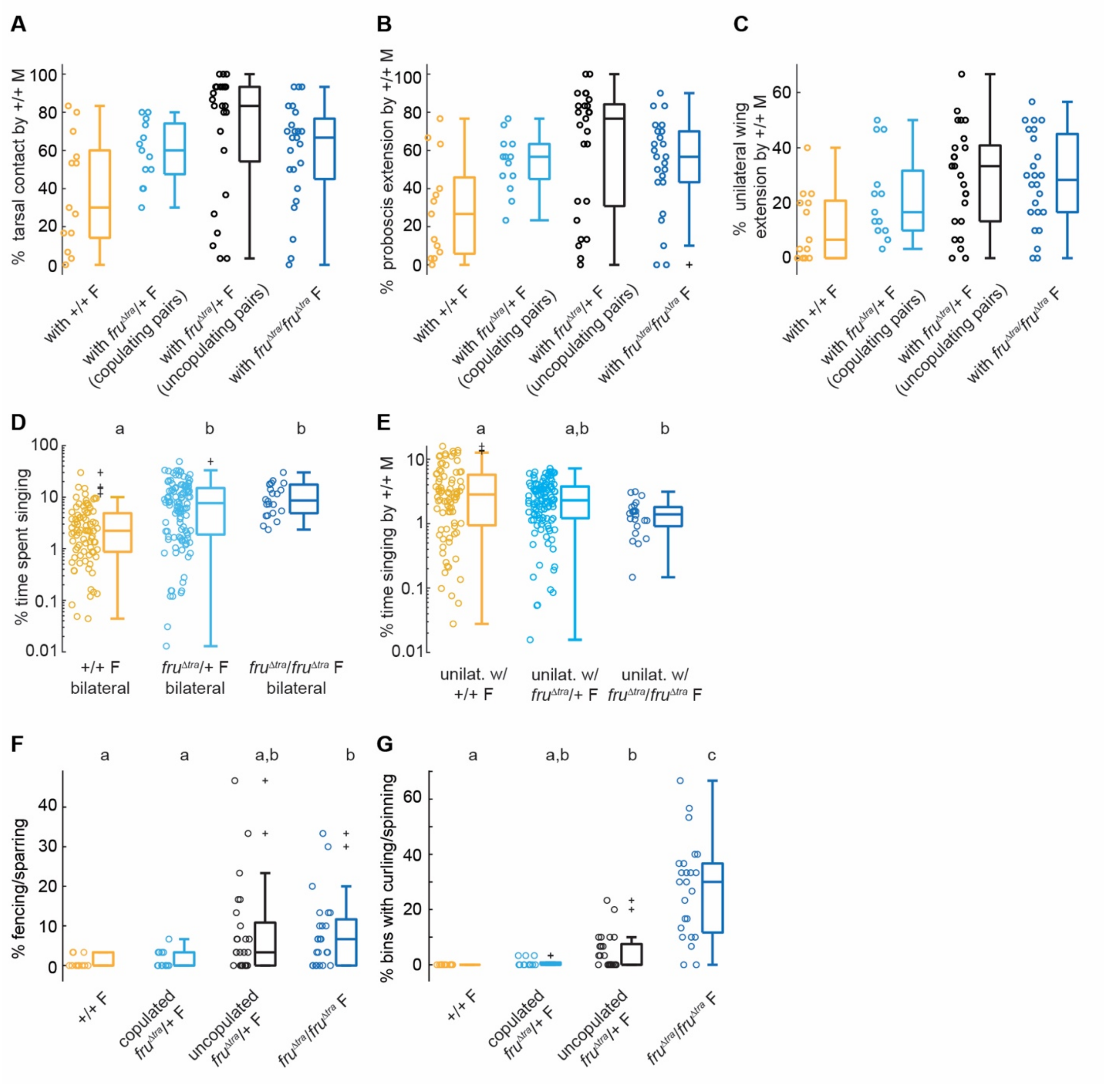

Fig. S6. Amount of courtship and aggressive behaviors in pairings with wild-type males. (**A-C**) Percentage of bins containing tarsal contact (**A**), proboscis extension (**B**), and unilateral wing extension (**C**) from the +/+ male toward females of each genotype. Even within *fru*$^{\Delta tra}$/+ females, males produced equal amounts of courtship regardless of whether the pair copulated. Kruskal-Wallis one-way ANOVA tests were significant for all 3 variables (**A-C**) but pairwise Wilcoxon rank sum tests did not meet significance after Bonferroni correction for multiple comparisons. n=13, 13, 25, 24 flies. (**D**) Percent courtship time occupied by bilateral song in pairings with +/+ males. Bilateral song in these pairings was produced solely by the female. (**E**) Same as (**D**) for unilateral song, which was produced solely by the +/+ male. Each dot represents one fly. n=83, 114, 22 flies. Statistical tests were Kruskal-Wallis one-way ANOVA followed by pairwise Wilcoxon rank sum tests with Bonferroni correction. (**F-G**) Percentage of bins

containing fencing/sparring (**F**) and curling/spinning (**G**) between females paired with +/+ males. There were no differences in the amount of these two behaviors in copulating vs. non-copulating *fru*$^{\Delta tra}$/+ females. n=13, 13, 25, 24 flies. Statistical tests were Kruskal-Wallis one-way ANOVA followed by pairwise Wilcoxon rank sum tests with Bonferroni correction.

Movie S1. *D. virilis* $fru^{\Delta tra}/fru^{\Delta tra}$ female taps, licks, and sings unilateral song to a +/+ female (white dot). The +/+ female responds with bilateral song.

Movie S2. *D. melanogaster* $fru^{\Delta tra}/+$ female taps, licks, and performs unilateral wing extensions directed toward a +/+ female (white dot). The wing extensions produce some sounds but not male-typical song.

Movie S3. *D. virilis* $fru^{\Delta tra}/fru^{\Delta tra}$ female sings bilateral song while duetting with a +/+ male (smaller fly).

Movie S4. Low-posture fencing between *D. virilis* $fru^{\Delta tra}/fru^{\Delta tra}$ female (larger fly) and +/+ male. The interaction begins with duetting, then the $fru^{\Delta tra}/fru^{\Delta tra}$ female turns to the male and approaches him with her front tarsi. She also produces a brief curling event and a few wing flicks. The male quickly resumes courting her once the aggression subsides.

Movie S5. Curling and spinning between *D. virilis* $fru^{\Delta tra}/fru^{\Delta tra}$ female (larger fly) and +/+ male. The interaction begins with the male courting the $fru^{\Delta tra}/fru^{\Delta tra}$ female and then the $fru^{\Delta tra}/fru^{\Delta tra}$ female initiates curling, in which she curls her abdomen toward the male and at times shoves him with it. The male appears to try to continue tapping and licking the $fru^{\Delta tra}/fru^{\Delta tra}$ female from behind, which results in the pair spinning around together. Duetting immediately resumes when the spinning bout ends.

REFERENCES AND NOTES

1. D. S. Manoli, P. Fan, E. J. Fraser, N. M. Shah, Neural control of sexually dimorphic behaviors. *Curr. Opin. Neurobiol.* **23**, 330–338 (2013).

2. K. C. Burtis, B. S. Baker, Drosophila doublesex gene controls somatic sexual differentiation by producing alternatively spliced mRNAs encoding related sex-specific polypeptides. *Cell* **56**, 997–1010 (1989).

3. E. Demir, B. J. Dickson, Fruitless splicing specifies male courtship behavior in *Drosophila*. *Cell* **121**, 785–794 (2005).

4. H. Ito, K. Fujitani, K. Usui, K. Shimizu-Nishikawa, S. Tanaka, D. Yamamoto, Sexual orientation in Drosophila is altered by the satori mutation in the sex-determination gene fruitless that encodes a zinc finger protein with a BTB domain. *Proc. Natl. Acad. Sci.* **93**, 9687–9692 (1996).

5. K. Kimura, T. Hachiya, M. Koganezawa, T. Tazawa, D. Yamamoto, Fruitless and doublesex coordinate to generate male-specific neurons that can initiate courtship. *Neuron* **59**, 759–769 (2008).

6. D. S. Manoli, M. Foss, A. Villella, B. J. Taylor, J. C. Hall, B. S. Baker, Male-specific fruitless specifies the neural substrates of *Drosophila* courtship behaviour. *Nature* **436**, 395–400 (2005).

7. Q. Peng, J. Chen, Y. Pan, From fruitless to sex: On the generation and diversification of an innate behavior. *Genes Brain Behav.* **20**, e12772 (2021).

8. L. C. Ryner, S. F. Goodwin, D. H. Castrillon, A. Anand, A. Villella, B. S. Baker, J. C. Hall, B. J. Taylor, S. A. Wasserman, Control of male sexual behavior and sexual orientation in Drosophila by the fruitless gene. *Cell* **87**, 1079–1089 (1996).

9. M. Salvemini, C. Polito, G. Saccone, Fruitless alternative splicing and sex behaviour in insects: An ancient and unforgettable love story? *J. Genet.* **89**, 287–299 (2010).

10. P. Stockinger, D. Kvitsiani, S. Rotkopf, L. Tirián, B. J. Dickson, Neural circuitry that governs *Drosophila* male courtship behavior. *Cell* **121**, 795–807 (2005).

11. K. S. Gill, A mutation causing abnormal courtship and mating behavior in males of Drosophila melanogaster. *Am. Zool.* **3**, 507 (1963).

12. G. Lee, M. Foss, S. F. Goodwin, T. Carlo, B. J. Taylor, J. C. Hall, Spatial, temporal, and sexually dimorphic expression patterns of the fruitless gene in the Drosophila central nervous system. *J. Neurobiol.* **43**, 404–426 (2000).

13. K. Usui-Aoki, H. Ito, K. Ui-Tei, K. Takahashi, T. Lukacsovich, W. Awano, H. Nakata, Z. F. Piao, E. E. Nilsson, J. Tomida, D. Yamamoto, Formation of the male-specific muscle in female *Drosophila* by ectopic fruitless expression. *Nat. Cell Biol.* **2**, 500–506 (2000).

14. R. C. Bertossa, L. van de Zande, L. W. Beukeboom, The fruitless gene in *Nasonia* displays complex sex-specific splicing and contains new zinc finger domains. *Mol. Biol. Evol.* **26**, 1557–1569 (2009).

15. D. A. Gailey, J.-C. Billeter, J. H. Liu, F. Bauzon, J. B. Allendorfer, S. F. Goodwin, Functional conservation of the fruitless male sex-determination gene across 250 Myr of insect evolution. *Mol. Biol. Evol.* **23**, 633–643 (2006).

16. K. Laohakieat, S. Isasawin, S. Thanaphum, The transformer-2 and fruitless characterisation with developmental expression profiles of sex-determining genes in *Bactrocera dorsalis* and *B. correcta*. *Sci. Rep.* **10**, 17938 (2020).

17. N. Meier, S. C. Käppeli, M. H. Niessen, J.-C. Billeter, S. F. Goodwin, D. Bopp, Genetic control of courtship behavior in the housefly: Evidence for a conserved bifurcation of the sex-determining pathway. *PLOS ONE* **8**, e62476 (2013).

18. M. Salvemini, R. D’Amato, V. Petrella, S. Aceto, D. Nimmo, M. Neira, L. Alphey, L. C. Polito, G. Saccone, The orthologue of the fruitfly sex behaviour gene fruitless in the mosquito *Aedes aegypti*: Evolution of genomic organisation and alternative splicing. *PLOS ONE* **8**, e48554 (2013).

19. T. Davis, J. Kurihara, D. Yamamoto, Genomic organisation and characterisation of the neural sex-determination gene fruitless (fru) in the Hawaiian species *Drosophila heteroneura*. *Gene* **246**, 143–149 (2000).

20. T. Davis, J. Kurihara, E. Yoshino, D. Yamamoto, Genomic organisation of the Neural sex determination gene fruitless (Fuu) in the Hawaiian species *Drosophila Siluestris* and the conservation of the Fru BTB protein-protein-binding domain throughout evolution. *Hereditas* **132**, 67–78 (2000).

21. T. Watanabe, Evolution of the neural sex-determination system in insects: Does fruitless homologue regulate neural sexual dimorphism in basal insects? *Insect Mol. Biol.* **28**, 807–827 (2019).

22. D. Yamamoto, K. Usui-Aoki, S. Shima, Male-specific expression of the fruitless protein is not common to all *Drosophila* species. *Genetica* **120**, 267–272 (2004).

23. R. Tanaka, T. Higuchi, S. Kohatsu, K. Sato, D. Yamamoto, Optogenetic activation of the fruitless-labeled circuitry in *Drosophila* subobscura males induces mating motor acts. *J. Neurosci.* **37**, 11662–11674 (2017).

24. N. S. Basrur, M. E. De Obaldia, T. Morita, M. Herre, R. K. von Heynitz, Y. N. Tsitohay, L. B. Vosshall, Fruitless mutant male mosquitoes gain attraction to human odor. *eLife* **9**, e63982 (2020).

25. B. Boerjan, J. Tobback, H. P. Vandersmissen, R. Huybrechts, L. Schoofs, Fruitless RNAi knockdown in the desert locust, Schistocerca gregaria, influences male fertility. *J. Insect Physiol.* **58**, 265–269 (2012).

26. B. Boerjan, J. Tobback, A. De Loof, L. Schoofs, R. Huybrechts, Fruitless RNAi knockdown in males interferes with copulation success in *Schistocerca gregaria*. *Insect Biochem. Mol. Biol.* **41**, 340–347 (2011).

27. E. Clynen, L. Ciudad, X. Bellés, M.-D. Piulachs, Conservation of fruitless' role as master regulator of male courtship behaviour from cockroaches to flies. *Dev. Genes Evol.* **221**, 43–48 (2011).

28. J. Xu, W. Liu, D. Yang, S. Chen, K. Chen, Z. Liu, X. Yang, J. Meng, G. Zhu, S. Dong, Y. Zhang, S. Zhan, G. Wang, Y. Huang, Regulation of olfactory-based sex behaviors in the silkworm by genes in the sex-determination cascade. *PLOS Genet.* **16**, e1008622 (2020).

29. E. J. Clowney, S. Iguchi, J. J. Bussell, E. Scheer, V. Ruta, Multimodal chemosensory circuits controlling male courtship in *Drosophila*. *Neuron* **87**, 1036–1049 (2015).

30. J. Kohl, A. D. Ostrovsky, S. Frechter, G. S. X. E. Jefferis, A bidirectional circuit switch reroutes pheromone signals in male and female brains. *Cell* **155**, 1610–1623 (2013).

31. I. M. A. Ribeiro, M. Drews, A. Bahl, C. Machacek, A. Borst, B. J. Dickson, Visual projection neurons mediating directed courtship in *Drosophila*. *Cell* **174**, 607–621.e18 (2018).

32. V. Ruta, S. R. Datta, M. L. Vasconcelos, J. Freeland, L. L. Looger, R. Axel, A dimorphic pheromone circuit in *Drosophila* from sensory input to descending output. *Nature* **468**, 686–690 (2010).

33. A. C. von Philipsborn, T. Liu, J. Y. Yu, C. Masser, S. S. Bidaye, B. J. Dickson, Neuronal control of *Drosophila* courtship song. *Neuron* **69**, 509–522 (2011).

34. Y. Ding, J. L. Lillvis, J. Cande, G. J. Berman, B. J. Arthur, X. Long, M. Xu, B. J. Dickson, D. L. Stern, Neural evolution of context-dependent fly song. *Curr. Biol.* **29**, 1089–1099.e7 (2019).

35. L. F. Seeholzer, M. Seppo, D. L. Stern, V. Ruta, Evolution of a central neural circuit underlies *Drosophila* mate preferences. *Nature* **559**, 564–569 (2018).

36. J.-C. Billeter, A. Villella, J. B. Allendorfer, A. J. Dornan, M. Richardson, D. A. Gailey, S. F. Goodwin, Isoform-specific control of male neuronal differentiation and behavior in *Drosophila* by the fruitless gene. *Curr. Biol.* **16**, 1063–1076 (2006).

37. S. F. Goodwin, B. J. Taylor, A. Villella, M. Foss, L. C. Ryner, B. S. Baker, J. C. Hall, Aberrant splicing and altered spatial expression patterns in fruitless mutants of Drosophila melanogaster. *Genetics* **154**, 725–745 (2000).

38. K. Usui-Aoki, Y. Mikawa, D. Yamamoto, Species-specific patterns of sexual dimorphism in the expression of fruitless protein, a neural musculinizing factor in *Drosophila*. *J. Neurogenet.* **19**, 109–121 (2005).

39. X. Song, J. L. Goicoechea, J. S. S. Ammiraju, M. Luo, R. He, J. Lin, S.-J. Lee, N. Sisneros, T. Watts, D. A. Kudrna, W. Golser, E. Ashley, K. Collura, M. Braidotti, Y. Yu, L. M. Matzkin, B. F. McAllister, T. A. Markow, R. A. Wing, The 19 genomes of Drosophila: A bac library resource for genus-wide and genome-scale comparative evolutionary research. *Genetics* **187**, 1023–1030 (2011).

40. H. T. Spieth, Courtship behavior in *Drosophila*. *Annu. Rev. Entomol.* **19**, 385–405 (1974).

41. A. Hoikkala, "Inheritance of male sound characteristics in *Drosophila* species" in *Insect Sounds and Communication: Physiology, Behavior, Ecology and Evolution* (CRC Press, 2005), pp. 167–177.

42. J. Donegan, A. W. Ewing, Duetting in *Drosophila* and Zaprionus species. *Anim. Behav*. **28**, 1289 (1980).

43. K. M. LaRue, J. Clemens, G. J. Berman, M. Murthy, Acoustic duetting in *Drosophila virilis* relies on the integration of auditory and tactile signals. *eLife* **4**, e07277 (2015).

44. E. J. Rideout, J.-C. Billeter, S. F. Goodwin, The sex-determination genes fruitless and doublesex specify a neural substrate required for courtship song. *Curr. Biol.* **17**, 1473–1478 (2007).

45. E. Vrontou, S. P. Nilsen, E. Demir, E. A. Kravitz, B. J. Dickson, Fruitless regulates aggression and dominance in *Drosophila*. *Nat. Neurosci.* **9**, 1469–1471 (2006).

46. S. P. Nilsen, Y.-B. Chan, R. Huber, E. A. Kravitz, Gender-selective patterns of aggressive behavior in *Drosophila melanogaster*. *Proc. Natl. Acad. Sci.* **101**, 12342–12347 (2004).

47. H. T. Spieth, Courtship behavior of endemic Hawaiian Drosophila. *Univ. Tex. Publ.* **6615**, 245–313 (1966).

48. H. T. Spieth, Mating behavior within the genus Drosophila (Diptera). *Bull. Am. Mus. Nat. Hist.* **99**, 7. (1952).

49. Y.-B. Chan, E. A. Kravitz, Specific subgroups of FruM neurons control sexually dimorphic patterns of aggression in *Drosophila melanogaster*. *Proc. Natl. Acad. Sci. U.S.A.* **104**, 19577–19582 (2007).

50. S. J. Certel, M. G. Savella, D. C. F. Schlegel, E. A. Kravitz, Modulation of *Drosophila* male behavioral choice. *Proc. Natl. Acad. Sci. U.S.A.* **104**, 4706–4711 (2007).

51. M. Wohl, K. Ishii, K. Asahina, Layered roles of fruitless isoforms in specification and function of male aggression-promoting neurons in *Drosophila*. *eLife* **9**, e52702 (2020).

52. R. Cook, ‘Lesbian’ phenotype of *Drosophila melanogaster*? *Nature* **254**, 241–242 (1975).

53. L. M. Ditch, T. Shirangi, J. L. Pitman, K. L. Latham, K. D. Finley, P. T. Edeen, B. J. Taylor, M. McKeown, *Drosophila* retained/dead ringeris necessary for neuronal pathfinding, female receptivity and repression offruitlessindependent male courtship behaviors. *Development* **132**, 155–164 (2005).

54. J. D. Clyne, G. Miesenböck, Sex-specific control and tuning of the pattern generator for courtship song in *Drosophila*. *Cell* **133**, 354–363 (2008).

55. C. Rezával, S. Pattnaik, H. J. Pavlou, T. Nojima, B. Brüggemeier, L. A. D. D’Souza, H. K. M. Dweck, S. F. Goodwin, Activation of latent courtship circuitry in the brain of *Drosophila* females induces male-like behaviors. *Curr. Biol.* **26**, 2508–2515 (2016).

56. S. Sethi, H.-H. Lin, A. K. Shepherd, P. C. Volkan, C.-Y. Su, J. W. Wang, Social context enhances hormonal modulation of pheromone detection in drosophila. *Curr. Biol.* **29**, 3887–3898.e4 (2019).

57. E. C. Pandolfi, H. M. Hoffmann, E. L. Schoeller, M. R. Gorman, P. L. Mellon, Haploinsufficiency of SIX3 abolishes male reproductive behavior through disrupted olfactory development, and impairs female fertility through disrupted GnRH neuron migration. *Mol. Neurobiol.* **55**, 8709–8727 (2018).

58. R. A. Veitia, Exploring the etiology of haploinsufficiency. *Bioessays* **24**, 175–184 (2002).

59. S. A. Morrill, A. Amon, Why haploinsufficiency persists. *Proc. Natl. Acad. Sci.* **116**, 11866–11871 (2019).

60. T. R. Shirangi, A. M. Wong, J. W. Truman, D. L. Stern, Doublesex regulates the connectivity of a neural circuit controlling *Drosophila* male courtship song. *Dev. Cell* **37**, 533–544 (2016).

61. K. Kimura, C. Sato, M. Koganezawa, D. Yamamoto, *Drosophila* ovipositor extension in mating behavior and egg deposition involves distinct sets of brain interneurons. *PLOS ONE* **10**, e0126445 (2015).

62. C. Zhou, Y. Pan, C. C. Robinett, G. W. Meissner, B. S. Baker, Central brain neurons expressing doublesex regulate female receptivity in *Drosophila*. *Neuron* **83**, 149–163 (2014).

63. K. Wang, F. Wang, N. Forknall, T. Yang, C. Patrick, R. Parekh, B. J. Dickson, Neural circuit mechanisms of sexual receptivity in *Drosophila* females. *Nature* **589**, 577–581 (2021).

64. E. J. Rideout, A. J. Dornan, M. C. Neville, S. Eadie, S. F. Goodwin, Control of sexual differentiation and behavior by the doublesex gene in *Drosophila melanogaster*. *Nat. Neurosci.* **13**, 458–466 (2010).

65. L. E. Sanders, M. N. Arbeitman, Doublesex establishes sexual dimorphism in the *Drosophila* central nervous system in an isoform-dependent manner by directing cell number. *Dev. Biol.* **320**, 378–390 (2008).

66. K.-I. Kimura, M. Ote, T. Tazawa, D. Yamamoto, Fruitless specifies sexually dimorphic neural circuitry in the *Drosophila* brain. *Nature* **438**, 229–233 (2005).

67. A. C. von Philipsborn, S. Jörchel, L. Tirian, E. Demir, T. Morita, D. L. Stern, B. J. Dickson, Cellular and behavioral functions of fruitless isoforms in *Drosophila* courtship. *Curr. Biol.* **24**, 242–251 (2014).

68. Y. Pan, G. W. Meissner, B. S. Baker, Joint control of *Drosophila* male courtship behavior by motion cues and activation of male-specific P1 neurons. *Proc. Natl. Acad. Sci.* **109**, 10065–10070 (2012).

69. R. T. Coleman, I. Morantte, G. T. Koreman, M. L. Cheng, Y. Ding, V. Ruta, A modular circuit architecture coordinates the diversification of courtship strategies in Drosophila. bioRxiv. 2023. www.biorxiv.org/content/10.1101/2023.09.16.558080v1.

70. V. Petrella, S. Aceto, V. Colonna, G. Saccone, R. Sanges, N. Polanska, P. Volf, L. Gradoni, G. Bongiorno, M. Salvemini, Identification of sex determination genes and their evolution in Phlebotominae sand flies (Diptera, Nematocera). *BMC Genomics* **20**, 522 (2019).

71. J. Cande, D. L. Stern, T. Morita, B. Prud'homme, N. Gompel, Looking under the lamp post: Neither fruitless nor doublesex has evolved to generate divergent male courtship in *Drosophila*. *Cell Rep.* **8**, 363–370 (2014).

72. T. M. Jinks, G. Calhoun, P. Schedl, Functional conservation of the Sex-lethal sex determining promoter, Sxl-Pe, in *Drosophila virilis*. *Dev. Genes Evol.* **213**, 155–165 (2003).

73. D. Chandler, M. E. McGuffin, J. Piskur, J. Yao, B. S. Baker, W. Mattox, Evolutionary conservation of regulatory strategies for the sex determination factor transformer-2. *Mol. Cell. Biol.* **17**, 2908–2919 (1997).

74. M. T. O'Neil, J. M. Belote, Interspecific comparison of the transformer gene of Drosophila reveals an unusually high degree of evolutionary divergence. *Genetics* **131**, 113–128 (1992).

75. R. J. Kulathinal, L. Skwarek, R. A. Morton, R. S. Singh, Rapid evolution of the sex-determining gene, transformer: Structural diversity and rate heterogeneity among sibling species of *Drosophila*. *Mol. Biol. Evol.* **20**, 441–452 (2003).

76. L. Suvanto, J. O. Liimatainen, A. Hoikkala, Variability and evolvability of male song characters in *Drosophila montana* populations. *Hereditas* **130**, 13–18 (1999).

77. E. Isoherranen, J. Aspi, A. Hoikkala, Variation and consistency of female preferences for simulated courtship songs in *Drosophila virilis*. *Anim. Behav.* **57**, 619–625 (1999).

78. D. J. Parker, A. Gardiner, M. C. Neville, M. G. Ritchie, S. F. Goodwin, The evolution of novelty in conserved genes; evidence of positive selection in the *Drosophila* fruitless gene is localised to alternatively spliced exons. *Heredity* **112**, 300–306 (2014).

79. K. Hoshijima, K. Inoue, I. Higuchi, H. Sakamoto, Y. Shimura, Control of doublesex alternative splicing by transformer and transformer-2 in *Drosophila*. *Science* **252**, 833–836 (1991).

80. S. J. Gratz, F. P. Ukken, C. D. Rubinstein, G. Thiede, L. K. Donohue, A. M. Cummings, K. M. O'Connor-Giles, Highly specific and efficient CRISPR/Cas9-catalyzed homology-directed repair in *Drosophila*. *Genetics* **196**, 961–971 (2014).

81. K. E. Kistler, L. B. Vosshall, B. J. Matthews, Genome engineering with CRISPR-Cas9 in the mosquito *Aedes aegypti*. *Cell Rep.* **11**, 51–60 (2015).

82. B. J. Arthur, T. Sunayama-Morita, P. Coen, M. Murthy, D. L. Stern, Multi-channel acoustic recording and automated analysis of *Drosophila* courtship songs. *BMC Biol.* **11**, 11 (2013).

83. D. Yamamoto, J.-M. Jallon, A. Komatsu, Genetic dissection of sexual behavior *in Drosophila melanogaster*. *Annu. Rev. Entomol.* **42**, 551–585 (1997).

84. J.-C. Billeter, S. F. Goodwin, Characterization of *Drosophila* fruitless-gal4 transgenes reveals expression in male-specific fruitless neurons and innervation of male reproductive structures. *J. Comp. Neurol.* **475**, 270–287 (2004).
